# Identification of a Theory-Practice Gap in the Education of Biomedical Scientists

**DOI:** 10.3389/bjbs.2024.12629

**Published:** 2024-06-12

**Authors:** Kathryn Dudley, David Matheson

**Affiliations:** ^1^ School of Life Sciences, Faculty of Science and Engineering, University of Wolverhampton, Wolverhampton, United Kingdom; ^2^ School of Nursing, Faculty of Education, Health and Wellbeing, University of Wolverhampton, Wolverhampton, United Kingdom

**Keywords:** biomedical scientist, professional practice, biomedical science graduates, patient outcomes, education and training

## Abstract

**Introduction:**

The Biomedical Scientist (BMS) role is established in healthcare, working in laboratory environments to provide diagnostic testing and to monitor treatment effects on a patients’ health. The profession is subject to several professional standards which highlight the importance of working in the best interests of the patient and service user. However, Biomedical Scientists have little or no patient contact. This study aimed to determine how Biomedical Scientists evidence that they meet the professional standards and support the achievement of patient outcomes.

**Materials and Methods:**

This study utilised a Delphi method to explore the opinions of professional stakeholders to determine whether there was consensus for how this professional group contributes to patient outcomes and offers evidence that they are working in the best interests of the patient. The qualitative 1st round of the study consisted of focus groups and interviews with staff and students on the BSc Biomedical Science awards, Professional, Statutory and Regulatory body (PSRB) representatives and Biomedical Scientists from the National Health Service (NHS). The first-round responses were analysed using thematic analysis which then generated attitude statements which participants scored using a 5-point Likert scale in the 2nd round. Consensus or divergence of opinion was determined based upon a 70% consensus level within each participant group and overall.

**Results:**

Following analysis of the 2nd round data, there was divergence of opinion across all stakeholders, with consensus rates being highest in the Biomedical Scientist group (72.7% of statements reached 70% consensus), followed by the student group (54.5% of statements reached 70% consensus) and lowest in the academic group (40.9% of statements reached 70% consensus).

**Discussion:**

This demonstrates a theory-practice gap in both the academic and student groups, suggesting that graduates are insufficiently prepared for their post-graduate role. This gap was particularly evident when discussing topics such as how Biomedical Scientists contribute to patient care, professional registration and working as part of the multi-disciplinary team (MDT). The identification of a theory-practice gap in the education of Biomedical Scientists is a novel finding, indicating that students may graduate with insufficient understanding of the Biomedical Scientist role.

## Introduction

Biomedical Scientists form a significant part of the healthcare scientific workforce within the UK healthcare system. There are 21,427 Biomedical Scientists registered in the UK, representing 7.6% of the 283,750 Health and Care Professions Council (HCPC) registered professionals within the UK [1]. Despite this, the role that Biomedical Scientists play in achievement of patient outcomes and how a Biomedical Scientist can evidence the impact of their role on those outcomes is not always explicit to students completing undergraduate Biomedical Science programmes. In many of the key biomedical science disciplines, Biomedical Scientists routinely experience minimal or no patient contact. However, the work carried out by Biomedical Scientists is an important part of patient care pathways and clinical decision making.

**FIGURE 1:**
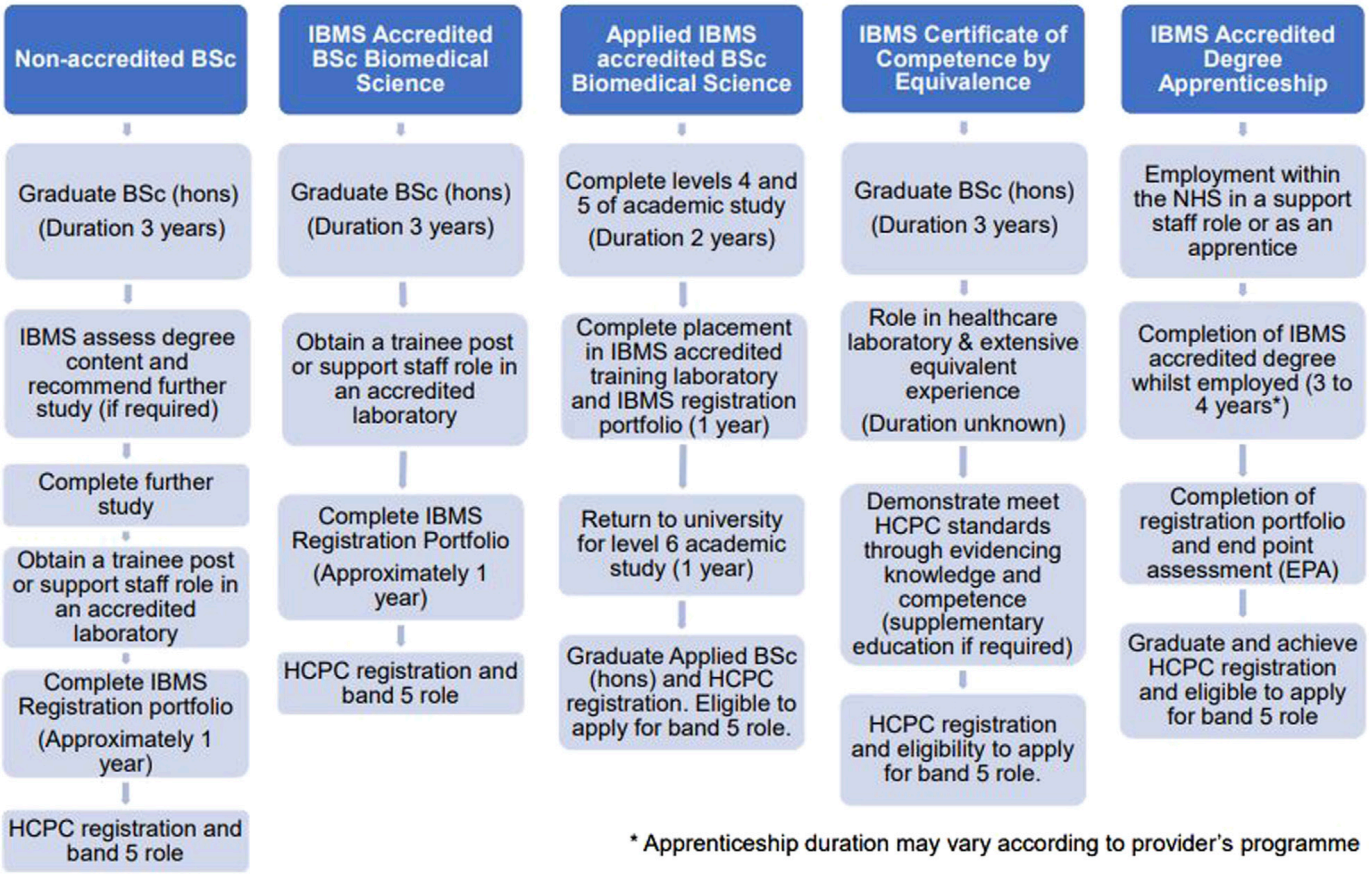
An overview of the different routes for achieving HCPC registration as a Biomedical Scientist [2–4].

The HCPC award the protected title of “Biomedical Scientist” to those who meet the necessary requirements to practice. Biomedical Scientists must successfully complete an Institute of Biomedical Science (IBMS) accredited degree (or equivalent qualification), a period of training in an approved laboratory, completion of the IBMS registration training portfolio and award of the Certificate of Competence [2, 3]. There are several different routes to achieve HCPC registration as a Biomedical Scientist (Figure 1), but availability of trainee Biomedical Scientist positions limits the number of graduates from IBMS accredited programmes who can join the register. Accredited Biomedical Science degrees must cover all key pathology disciplines and the academic requirements to become HCPC registered. Increasingly, HCPC registration for Biomedical Scientists involves the completion of a year-long placement or an integrated degree apprenticeship. This demonstrates that education providers play a pivotal role in fostering student understanding of the Biomedical Scientist role and preparing students for their post-graduate roles.

### Understanding the Wider Context of the Biomedical Scientist Role

Biomedical Scientists must comply with the HCPC standards of proficiency (SoPs) associated with their profession [5] and also with the standards of conduct, performance and ethics [6]. The standards of conduct, performance and ethics are currently under review with a new version being implemented in September 2024 [7]. The HCPC regulate 15 health professions, including dieticians, physiotherapists and occupational therapists [8]. The standards recognise that the range of different professions regulated by the HCPC may have differences in their scope of practice. As a result, some professions are acknowledged to work with patients, others with clients and some with service users. The standards of proficiency for Biomedical Scientists use the phrase *“service user”* when describing the groups that use or are affected by the Biomedical Scientist role [5]. For Biomedical Scientists, service users can be regarded as patients and also as clinical staff utilising the laboratory service.

For students and trainee Biomedical Scientists, it can be challenging to comprehend the importance of the individual patient as the ultimate service user due to a lack of patient contact included as part of their role. This can also provide a challenge for academic staff who are attempting to communicate this key concept to students on a Biomedical Science degree programme, many of whom will lack clinical exposure due to the competitive nature of NHS placement availability. This lack of clinical exposure can provide difficulties when relating theoretical ideas delivered in a taught session to real-world scenarios, which is often described as a theory-practice gap.This is exacerbated because academics delivering Biomedical Science programmes represent a diverse range of backgrounds, including both practitioners with firsthand NHS experience and researchers who may have little knowledge of the clinical laboratory environment. This differs from other fields of healthcare, for example, nursing, where most academics have firsthand experience of the role [9]. Furthermore, only some of the graduates of Biomedical Science awards aspire to work as a Biomedical Scientist, which provides a challenge for course design and ensuring appropriate course content that will interest the range of students which study Biomedical Science.

Applying the professional standards of the HCPC can be challenging for Biomedical Scientists. For example, HCPC standard of proficiency 2.5 states that Biomedical Scientists will “respect and uphold the rights, dignity, values and autonomy of service users, including their role in the assessment, diagnostic, treatment and/or therapeutic process” [5]. For Biomedical Scientists and academic teams preparing Biomedical Science students for practice, it can be challenging to recognise the service users’ values due to a lack of opportunities for patient interaction. It is possible that students on Biomedical Science degree courses lack practice opportunities which would allow them to contextualise how their work impacts the patient and service user. Further examples of the key HCPC standards which students may find difficult to contextualise are provided in Table 1.

**TABLE 1:** An overview of a selection of the key patient centred standards and guidelines in the literature associated with the Biomedical Scientist role [5, 6, 10]. These standards may be challenging for students to comprehend without clinical laboratory experience. Copyright permissions have been obtained from the HCPC and IBMS to reproduce these standards and professional guidelines.

HCPC standards of proficiency for biomedical scientists [5]	
Standard number	Standard
2.2	“Promote and protect the service user’s interests at all times”
2.5	“Respect and uphold the rights, dignity, values and autonomy of service users, including their role in the assessment, diagnostic, treatment and/or therapeutic process”
2.6	“Recognise that relationships with service users, carers and others should be based on mutual respect and trust, maintaining high standards of care in all circumstances”
7.4	“Work with service users and/or their carers to facilitate the service user’s preferred role in decision-making, and provide service users and carers with information they may need where appropriate”
8.1	“Work in partnership with service users, carers, colleagues and others”
8.12	“Understand the need to engage service users and carers in planning and evaluating diagnostics and assessment outcomes to meet their needs and goals”
8.13	“Demonstrate awareness of the impact of pathology services on the service user care pathway”
11.5	“Evaluate care plans or intervention plans using recognised and appropriate outcome measures, in conjunction with the service user where possible, and revise the plans as necessary”
14.1	“Understand the need to maintain the safety of themselves and others, including service users, carers and colleagues”
15.3	“Empower and enable individuals (including service users and colleagues) to play a part in managing their own health”

HCPC standards of conduct, performance and ethics [6]	
Standard number	Standard
1.1	“You must treat service users and carers as individuals, respecting their privacy and dignity”
1.2	“You must work in partnership with service users and carers, involving them, where appropriate, in decisions about their care, treatment or other services to be provided”
2.2	“You must listen to service users and carers and take account of their needs and wishes”
6.1	“You must take all reasonable steps to reduce the risk of harm to service users, carers and colleagues as far as possible”

IBMS good professional practice in biomedical science [10]	
Guideline number	Relevant guideline
Code of Conduct 1.2	“Maintain the highest standards of professional practice and act in the best interests of patients, the service and other professionals”
Code of Conduct 3.2	“Take action without delay if patient safety or service delivery is at risk according to local and national “whistleblowing” guidelines”
Code of Conduct 3.3	“Treat all patients, service users and colleagues respectfully and equally without any discrimination or prejudice that could hurt or embarrass”
1. Professional practice	“As a biomedical science professional, you have a duty of care (directly or indirectly) to the patient and must always ensure their safety and wellbeing”
11. Communication	“You understand the need to provide service users or people acting on their behalf with the information necessary to enable them to make informed decisions”
12. Partnerships and cooperation	“You will work in partnership and cooperation with service users, carers, colleagues and others for the benefit of the patient and service”

The IBMS guidelines for good professional practice and conduct in Biomedical Science are clear about the significance of the patient for Biomedical Science professionals, stating that “you will work in partnership and cooperation with service users, carers, colleagues and others for the benefit of the patient and service” [10]. Whilst this guideline is of key importance for Biomedical Scientists and students aspiring to this as a future career, the way in which Biomedical Scientists relate to this guideline and how students can develop an understanding of it through their education and professional experience requires further exploration.

### The Role of Pathology Laboratories in Patient Outcomes

There is no commonly accepted universal definition of patient outcomes, but these usually represent a change in health of a group or an individual due to an intervention [11]. In most cases, these outcomes are centred around a particular disease and assessment involves determining symptoms and clinical presentation [12]. However, this disease-centred model fails to focus upon key measures from the patients’ perspective, such as health status and quality of life. The focus upon disease-centred metrics could provide a further challenge for Biomedical Scientists to recognise the importance of the patient as service user, as described in the HCPC standards [5, 6] and the IBMS guidelines [10]. For Biomedical Scientists and students aspiring to this career, the role that Biomedical Scientists have in supporting achievement of patient outcomes has not been defined in professional literature.

It is well established that pathology services have a significant impact upon healthcare and patient care pathways. The work carried out by pathology services is involved during the lives of most patients, with a role from pre-natal screening and throughout the patient’s lifetime. As a result, pathology services cost the NHS between £2.5 and £3 billion per annum and represents 1.5%–3% of overall NHS expenditure [13]. Those practicing within Biomedical Science are familiar with the statistic suggesting that more than 70% of NHS diagnoses depend upon pathology test results [14–16]. This suggests that Biomedical Scientists are involved in diagnostic testing of a significant number of specimens and through this, impact upon a significant number of patient care pathways.

Incorrect or inaccurate laboratory test results or inappropriate result reporting is known to negatively impact patient care, resulting in unwarranted diagnostic testing, inappropriate treatment, patient anxiety and even death [17]. However, the role that Biomedical Scientists play within this process has not been explored, since much of the literature focuses upon pathology services as a whole. The role that Biomedical Scientists play within patient outcomes is challenging to measure due to the complex and integrated nature of healthcare. To provide a benefit to patient care, laboratory testing should be carried out at the right time, on the right patient and actioned in an appropriate time frame [17–20]. Through the impact of pathology services on patient care, the Biomedical Scientist role is key for supporting other healthcare professionals to fulfil their roles effectively. However, to what degree the Biomedical Scientist role impacts upon outcomes for the individual patient has not been explored.

### Putting the Patient at the Heart of the Biomedical Scientist Role

In the UK, several key NHS values are defined within the NHS constitution. The NHS constitution states “the patient will be at the heart of everything the NHS does” [21]. This includes involving patients, family members and carers in health and treatment decisions. Despite these concepts being a central part of NHS policy, the nature of the Biomedical Scientist role and the lack of direct patient contact means that evidencing these characteristics can be challenging. To date, the literature has not defined what it means to put the patient at the heart of the Biomedical Scientist role. For many healthcare professions, this is aligned to person-centred care (PCC). However, providing a definition of PCC that is applicable to the Biomedical Scientist role has proved challenging. Definitions of PCC are mainly focused upon clinicians, rather than recognising the role played by other healthcare professionals, but suggests that clinicians must be honest and respectful, demonstrate empathy and be compassionate [22]. Based on this definition, there is no reason why other healthcare professionals cannot be seen to evidence PCC in their practice.

For laboratories to practise PCC, it is necessary for them to investigate all stages of the total testing process (including sample collection, test requesting, reporting and actioning results) that may negatively impact patient outcomes [17]. The assumption is often made that through reporting laboratory results and the subsequent impact of these on patient care pathways, that optimising laboratory processes and achieving accurate results in a timely fashion are key to positively influence patient care. However, without appropriate and timely clinical action following laboratory testing, there is little clinical benefit to carrying out these tests [23].

According to McCormack and McCance [24], PCC is developed via the establishment of a therapeutic relationship between care providers, patients and carers. For Biomedical Scientists, developing these therapeutic relationships with patients and their carers is challenging due to a lack of interaction with the patient. However, providing high-quality care which is patient focused is considered an essential aspect of the Biomedical Scientist role by most in the profession. Despite this, key terms which are often used when describing the Biomedical Scientist role, such as “putting the patient at the centre” or “working in the best interests of the patient” have not been fully explained within the literature. Although the HCPC standards define the requirements of Biomedical Scientists with respect to service users and carers, students completing a Biomedical Science degree without clinical laboratory experience may find these standards difficult to interpret.

### Aims and Objectives

This study aimed to identify how stakeholders of the Biomedical Scientist role recognise that Biomedical Scientists are meeting the HCPC standards and other professional guidelines to support the achievement of patient outcomes.

As a result, this study aimed to address the following research questions through the recruitment of stakeholders of the Biomedical Scientist role:

1. Is there consensus amongst stakeholders upon how the role of the Biomedical Scientist influences patient outcomes?
2. Do stakeholders have a common understanding of how Biomedical Scientists might demonstrate that they are working to achieve patient outcomes?
3. How do stakeholders consider the importance of achieving patient outcomes within the Biomedical Scientist role?
4. How do stakeholders recognise a Biomedical Scientist who is working to support the achievement of patient outcomes?

The stakeholders were Biomedical Scientists working within the NHS, academic staff teaching on the BSc Biomedical Science degree programmes, representatives from the professional and statutory bodies and final year students on the BSc Biomedical Science awards.

## Materials and Methods

### The Delphi Methodology

This study aimed to identify how stakeholders of the profession recognised the Biomedical Scientist role in achieving patient outcomes. These concepts had not previously been defined in the literature. To do this, a modified Delphi methodology was used. Delphi is a consensus methodology whereby experts are invited to participate with a view to determining the consensus level on a topic. This is underpinned by the concept that the opinion of a group is seen as more beneficial than that of a single individual [25]. The Delphi methodology is useful to generate ideas and understand complex topics and is particularly useful in fields which lack previous data, which suggested it was valuable in this case [26].

**FIGURE 2:**
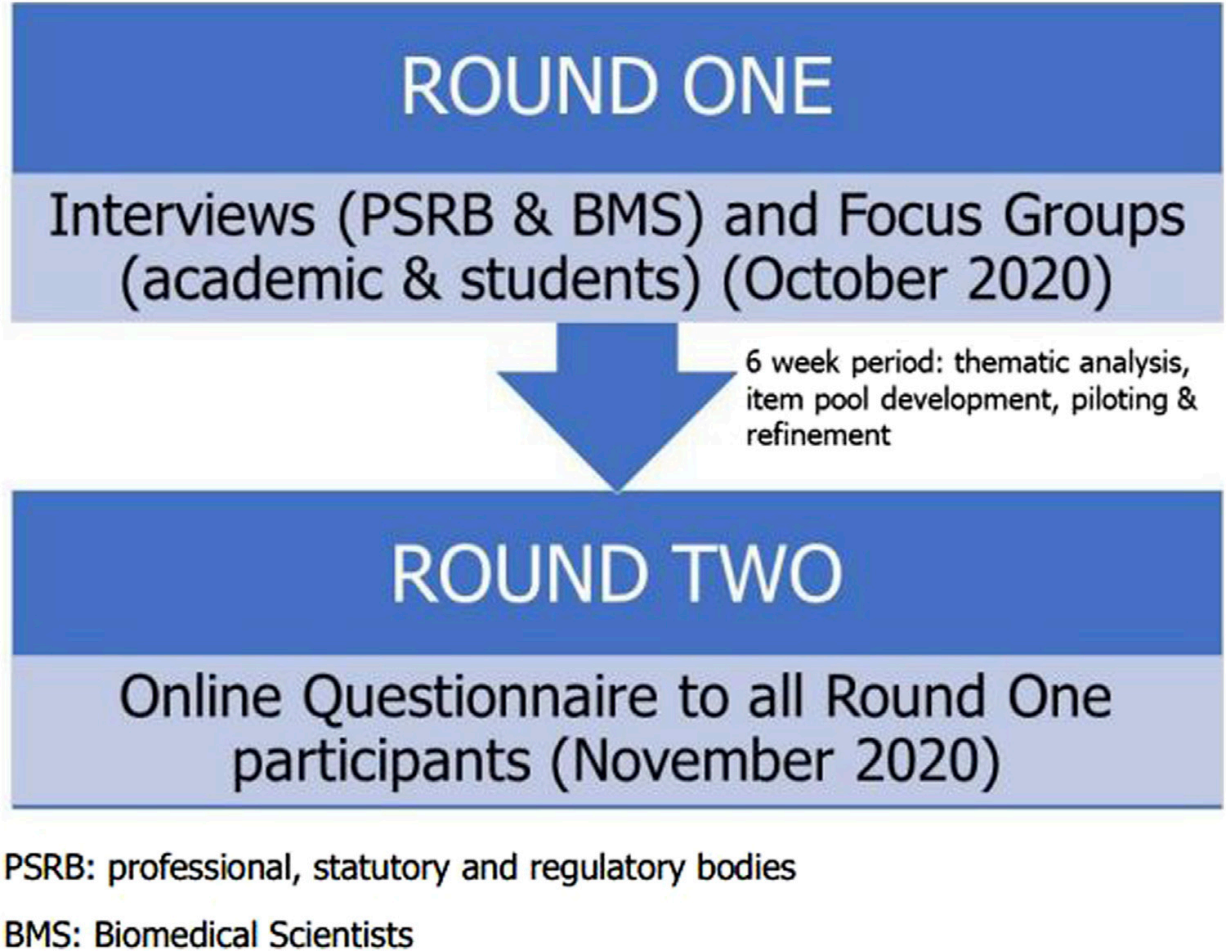
A schematic representation of the design of this Delphi study.

The study considered whether there was stakeholder consensus in key aspects of the Biomedical Scientist role and was carried out across two rounds. The qualitative first round involved semi-structured interviews and focus groups where participants were presented with a vignette and questions relating to role of the Biomedical Scientist within that case (Supplementary Data Sheet S1). The first-round data was analysed using thematic analysis which was used to produce a series of statements for the second round of the study. The second round was quantitative where participants scored their agreement with statements generated following the thematic analysis using a Likert scale (Supplementary Data Sheet S2). It was important to present statements in the 2nd round which utilised the participants’ phrasing with minimal editing to minimise researcher bias [27]. In a traditional Delphi study, there usually follows 2–4 subsequent rounds, but this modified Delphi methodology employed only a single subsequent round because the study was concerned with determining whether consensus existed and did not aim to necessarily achieve consensus. An overview of the study design can be found in Figure 2.

### Ethical Approval

This study was conducted during height of the COVID-19 pandemic and, as a result, involved the use of virtual data collection methods to comply with the requirements of the ethics committees that reviewed the study. Ethical approval was obtained from the Faculty of Education, Health and Wellbeing (FEHW) ethics committee, along with the Life Sciences ethics committee where the participating staff and students were recruited. Ethical approval was obtained from the NHS Health Research Authority (HRA) (IRAS ID: 273632, REC reference 20/LO/0675). HRA approval for the study was made subject to the 2nd round questionnaire being sent to the committee once it was developed. The Research and Development (R&D) department at the participating NHS Trust also confirmed capacity for the study.

### Round One

In the first round of the study, academics (*n* = 5) and final year students (*n* = 7) involved with the BSc Biomedical Science programmes and Biomedical Scientists (*n* = 5) at a local NHS Trust were invited to attend a series of virtual focus groups. Focus groups were considered a suitable data collection method for round one, as these are beneficial for generating rich, high-quality data [28]. Policy influencing representatives from professional, statutory and regulatory body (PSRB) organisations, such as the HCPC, IBMS and Department of Health were invited to attend one-to-one online or telephone interviews. PSRB organisations are external bodies which accredit and approve degree courses associated with professional qualifications. PSRB representatives (*n* = 2) were invited to attend interviews rather than a focus group to provide a more appropriate data collection method for this smaller stakeholder group.

Stakeholders were presented with a carefully designed case study which was accessible regardless of discipline and professional background and required prompt action from a Biomedical Scientist. The questions presented in the focus groups and interviews were developed and refined following an interview with a non-HCPC registered academic colleague. One of the strengths of Delphi methodology is that it allows for verification of findings through the use of both qualitative and quantitative data, where the quantitative data can be used to support the conclusions drawn from the qualitative data.

Audio recordings of the focus groups and interviews were made and stored in an anonymised format. These were transcribed shortly after the interview or focus group had taken place to ensure accuracy of the transcripts [29]. The transcription utilised a verbatim transcription approach to accurately record the discussions which had taken place. Participant responses were recorded without correcting sentence structure to capture the voice of the participants. The final stage of transcription involved addition of punctuation, which was checked for accuracy by replaying the recordings.

#### Thematic Analysis

Analysis of the 1st round data was carried out using content analysis techniques, utilising the Braun and Clarke [29] thematic analysis methodology, which is a flexible and accessible methodology. The initial step in thematic analysis involved becoming familiar with the transcripts through re-reading to identify the most obvious themes. This involved developing an understanding of the data through reading the words critically and analytically. Manual complete coding was carried out to ensure that all relevant data was considered. Coding involved identifying aspects of the data that related to the initial research question and the codes generated provided a label for a feature of the data that may have been of interest [29]. The codes were designed to be concise and capture the essence of the data. Codes were based upon the discussion points generated by the participants but were often defined in different terminology due to the need to be informative without the use of the data set.

As the codes were identified from the transcripts, patterns within the data began to emerge. Themes were identified when commonalities and areas of overlap became clear within the codes. Themes were defined as patterns seen across the data which had a clear central organising concept and also captured the principle of the participant’s experiences [29, 30]. This process required constant revision as new themes and relationships between themes were identified. Once the themes were identified, these themes were the basis for the attitude statements used in the 2nd round of the study.

### Round Two

In round two, participants were given a number of statements that they scored using a 5-point Likert scale. These statements were developed following the thematic analysis carried out at the end of the 1st round. Following the 1st round, an item pool was generated which consisted of numerous statements, according to the protocols outlined by Oppenheim [31] and Hicks [32]. These statements were developed using quotes provided in round one. In total, 94 attitude statements were development, including 47 positive and negative paired statements. To minimise satisficing, the attitude statements consisted of pairs of positive and negative statements. Satisficing involves research participants selecting responses they perceive as acceptable, agreeing with positive statements, or responding to every statement in a similar way [33, 34]. Statements were written to address a single opinion and to be clear and explicit to avoid confusion.

The 94 attitude statements that were initially developed were reduced using an item pool reduction. Traditionally, Delphi studies often experience high levels of attrition between questionnaire rounds; therefore, it was necessary to include an appropriate number of statements which would not be considered excessive or repetitive [25, 35]. The original 94 draft attitude statements were shared with 15 academics in the Department of Biomedical Science and Physiology who had not participated in the 1^st^ round of the study. The decision to pilot this questionnaire with a group of academics is a recognised limitation of the study. The implications of this are discussed further in the discussion. Through this piloting exercise, statements with low power to discriminate between high and low scoring groups were removed [32]. This resulted in 22 pairs of statements which were included in the 2nd round questionnaire (Supplementary Data Sheet S2). There was an additional one ranking question which was focused around identifying key priorities within the Biomedical Scientist role.

Participants were asked to score the attitude statements using a 5-point Likert scale, consisting of strongly agree, agree, neither agree nor disagree, disagree and strongly disagree. If a participant felt that they could not express an opinion on a topic for whatever reason, they were asked to omit their response. Before distribution, randomisation of the statements was performed to ensure it was not clear that positive and negative statements were present. This was designed to reduce satisficing [32].

#### Consensus

The aim of the second round was to determine whether consensus amongst stakeholders of the Biomedical Scientist role existed when considering the role of the Biomedical Scientist in patient outcomes. In a Delphi study, consensus is defined as a participant agreeing with a particular statement, which demonstrates both a group opinion and a level of participant agreement [25, 36]. Consensus levels differ in the literature, but usually range from 51% up to 80% [27, 37]. This study adopted a consensus level of 70%, despite the wide-ranging consensus levels reported in the literature. This meant that consensus was reached only if 70% or more of participants agreed or strongly agreed and disagreed or strongly disagreed with a particular statement. This 70% consensus level has been widely employed within the literature [25, 38].

### Recruitment Strategy

Two focus groups were carried out at a large West Midlands university. The first focus group involved final year students (*n* = 7), five on the BSc Biomedical Science and two on the BSc Applied Biomedical Science programmes. The second focus group involved academic staff (*n* = 5) delivering the Biomedical Science programmes. Final year students were invited to participate regardless of whether they had obtained experience in a clinical laboratory and some participants lacked clinical laboratory experience whilst others had returned from a placement year and would be able to apply for HCPC registration upon graduation. Biomedical Scientists employed at a local NHS Trust (*n* = 5) were also invited to attend a focus group through a gatekeeper, but the logistics of arranging a focus group for this group proved challenging and participants expressed a preference for a one-to-one interview. Representatives of 3 different PSRB organisations were invited to participate in one-to-one interviews held electronically, with both participants in this group representing the IBMS. Participants received a recruitment letter, a copy of the participant information sheet which provided an overview of the study and a link to the online consent form via email.

### Sample Size

The Delphi methodology has no universally accepted minimum number of participants [25]. However, the focus groups initially planned to include 6–8 participants. Possibly because of the COVID-19 pandemic, the number of participants recruited for the study was low but deemed acceptable if focus groups rather than questionnaires were used in the 1st round. Focus groups with too many attendees can become difficult to manage and it can be challenging for individuals to make their point known [28]. Two virtual focus groups were carried out in the 1st round via Microsoft Teams. One of these focus groups recruited 7 students from the BSc Biomedical Science programmes whilst the other recruited 5 academic staff from the same award. One-to-one interviews were carried out with 2 PSRB representatives (both representing the same organisation) and 5 HCPC registered Biomedical Scientists, resulting in a total of 19 participants recruited in the first round. An overview of the participant’s experience of the Biomedical Scientist role is presented in Table 2.

**TABLE 2:** The chosen method of data collection used in round one of the study by participant. As far as possible, participant codes, any relevant experience and their chosen discipline are presented unless it was considered necessary to withhold this to maintain anonymity.

Participant group	Data collection method	Participant codes	Experience of the BMS role	Discipline
Biomedical Scientists (n = 5)	One-to-one interviews	BS1 BS2 BS3 BS4 BS5	1 year in NHS <1 year in NHS >25 years in NHS >25 years in NHS 5–10 years in NHS	Haematology Haematology Immunology Biochemistry Haematology
Academics (*n* = 5)	Focus group	AC1 AC2 AC3 AC4 AC5	HCPC registered No experience as a BMS No experience as a BMS HCPC registered Completed NHS placement	Not disclosed to preserve anonymity
Students (*n* = 7)	Focus group	S1 S2 S3 S4 S5 S6 S7	Not completed placement Not completed placement Not completed placement Not completed placement NHS placement student NHS employment (non-BMS) NHS placement student	N/A N/A N/A N/A Biochemistry Immunology Histology
PSRB (*n* = 2)	One-to-one interviews	PB1 PB2	Experience as a BMS Experience as a BMS	Not disclosed to preserve anonymity
Total Participants		19		

Although recruitment of 19 participants for the first round of the study was lower than anticipated, utilising qualitative data collection methods in the 1st round allowed for a deep approach to data acquisition [36]. One advantage of the Delphi methodology is that smaller participant numbers are acceptable, with panel sizes of between 10 and 15 participants recognised as acceptable [39]. As the research was conducted during the COVID-19 pandemic, this may have contributed to the lower-than-expected number of participants.

### Response Rates

Due to the use of a gatekeeper at the participating NHS Trust who sent out invitations to prospective participants, the response rate for the 1st round of the study is unknown. Participants who had consented to participate in the 1st round received a link to the 2nd round JISC Online Surveys questionnaire via email. Respondents did not provide any identifiable information in the 2nd round, but instead selected their participant group meaning that it was not possible to identify individual participants. A final reminder email was sent a week before the deadline to participate. This resulted in 16 responses received in round 2 and response rates for each group are shown in Table 3. Unfortunately, both PSRB representatives were lost in the 2nd round of the study. The 2nd round response rate of 92.1% was considered acceptable for Delphi methodology, where it is commonly accepted that response rates exceeding 70% are required to maintain rigour [25].

**TABLE 3:** Response rates and the number of participants recruited for each round of the study.

Participant group	Participants in round one	Participants in round two
HCPC Registered Biomedical Scientists	5	4 (80% response rate)
Academics on BSc Biomedical Science programme	5	5 (100% response rate)
Students on BSc Biomedical Science	7	7 (100% response rate)
PSRB Representatives	2	0 (0% response rate)
**Total number of participants**	**19**	**16**
**Overall Response rate**	**35/38 = 92.1% Response rate**	

## Results

### Round 1

**FIGURE 3:**
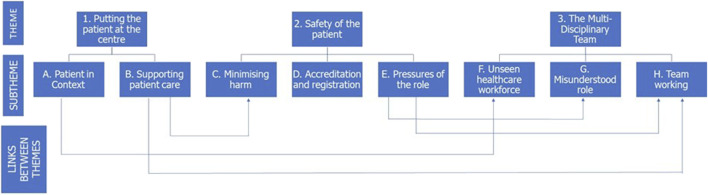
Diagrammatic representation of the three themes and subthemes identified following thematic analysis of the 1st round data. Where overlap between the themes exist, this is indicated by arrows.

In the 1st round, participation involved contribution in focus groups or semi-structured interviews. Following this qualitative round, a thematic analysis was carried out and a thematic map was devised (Figure 3). This thematic map identified the link between several of the themes and sub-themes. Illustrative quotes, previously presented in the doctoral thesis [40] from which the present article is drawn, are provided which support the thematic analysis and are representative of the voice of the participants.

### Round One Findings to Support the Presence of a Theory-Practice Gap

#### Putting the Patient First

For many participants, putting the patient at the centre was considered an essential aspect of the Biomedical Scientist role, despite this term not being defined in the literature. The Biomedical Scientist role was seen as essential for patient care and fulfilling this role in patient care was considered an essential motivation for aspiring Biomedical Scientists.

Participants highlighted the importance of the patient for Biomedical Scientists and why it was necessary to support achievement of outcomes for their patients. One of the participants in the Biomedical Scientist group stated how every specimen represents a patient:

“*If you don’t see the patient at the end of it you almost think ‘well I’ll take my time over that, I won’t worry about doing it particularly quickly’, whereas, you know, you could have a patient sat in the emergency department or the theatre waiting for a particular result and they’re not getting it*.”—Biomedical Scientist BS4.

This was also reflected by one of the participants in the student group:

*“When you’re looking at just the lab results, as a Biomedical Scientist so you need to have that in mind, that there is a patient on the other side of that test result and it … ultimately, what you decide, well … will affect their lives.”*—Student S4.

Participants frequently used expressions such as “putting the patient first,” but how this can be defined for Biomedical Scientists has not been previously discussed. One participant in the student group who had completed an NHS laboratory placement stated that focusing upon the importance of the patient motivated them to join the profession:

*“You’re becoming a Biomedical Scientist because you want to help people, particularly in a hospital setting because you’re going into pathology to run patient tests. You’ve kind of got to have a reason for doing that.”*—Student S5, NHS placement student.

This was further reflected by a Biomedical Scientist participant who spoke passionately about their role in patient care:

*“So that’s why I took the role as a Biomedical Scientist, because in my role, I’m still helping the patient and I’m still helping to find out what’s wrong with the patient.”*—Biomedical Scientist BS5.

Following the interview questions, one PSRB representative discussed how Biomedical Scientists can demonstrate that they are working to achieve patient outcomes:

*“OK. I found those quite difficult. I feel quite ashamed of myself because you’d think, after all these years of me talking to people (pause) but it’s just one of those things you take for granted.”*—PSRB representative PB1.

The fact that this key concept associated with the Biomedical Scientist role was considered as “taken for granted” was an important finding for this study.

Another PSRB representative remarked that there were challenges associated with appreciating the patient’s perspective as part of the Biomedical Scientist’s role due to minimal patient contact.

*“There’s not many Biomedical Scientists now who do phlebotomy, so they’ve got a lack of understanding of what patients are going through. When I trained, we went round, especially as trainees, to bleed the patients and you get a huge understanding of what patients are actually going through that I don’t think modern Biomedical Scientists get.”*- PSRB representative PB2.

Amongst the academic stakeholders, there were some conflicting opinions expressed by participants which did not align with those of other stakeholder groups. When discussing whether it was necessary to focus upon the patient’s best interests, the pressurised nature of the laboratory environment was noted by one HCPC-registered participant and how this could detract from the necessary focus upon patient outcomes:

*“When you get that many specimens, it’s difficult to devote … the same amount of attention to each one, but yeah, you’re striving to have the patient’s interests at heart and their outcomes … I think the size of modern labs almost, they’ve turned into sausage factories if you will … Patients are almost viewed as a number.”*—Academic AC4, HCPC-Registered academic.

This was further reflected by another academic participant, who stated the following when discussing whether patient outcomes were considered a crucial aspect of the Biomedical Scientist role:

*“I still think a Biomedical Scientist would do the tests accurately, even if they didn’t think about the patient, you’d hope, because that’s the scientist part of it.”*—Academic AC2.

Across the student and practising Biomedical Scientist groups, stakeholders felt the importance of the patient was an essential motivator for the role, but some of the academic stakeholders felt the high-pressured nature of the role detracted from this.

#### Professional Registration and Accreditation

Participants also discussed why professional registration, accreditation of laboratories by relevant organisations and adherence to guidelines provided by the IBMS [10] and the importance of the HCPC standards [5–7] for ensuring high quality care:

*“I wouldn’t want myself or a family member to come into a hospital that doesn’t have these regulatory bodies or these legal aspects to them because then I wouldn’t feel safe.”*—Biomedical Scientist BS5.

This was further reflected by comments from one of the PSRB representatives when asked how to recognise a Biomedical Scientist who is failing to focus upon patient outcomes:

*“It can seriously affect the patient and also contravenes the regulatory, the regulations by which we are registered which can lead to disciplinary action and, people being taken off the register so there are professional consequences as well as consequences for the patient.”*—PSRB Representative PB1.

#### Pressures of the Role

Comments related to the pressures of the role existed only in the academic group and not in the student or Biomedical Scientist groups. Some of the academic participants discussed the pressures associated with turn-around times and the volume of samples received in the laboratory, recognising that these could prevent Biomedical Scientists focusing upon the individual patient.

*“Yeah, I feel that in the lab that I came from, that if you imagined those two poles apart, quantity and quality, I feel it’s moving slightly from the quality towards the quantity … As a result, the quality of the work is not as good as it should be because they are focusing upon quantity.”*—HCPC Registered Academic AC4.

This was reflected by another academic participant who stated:

*“You’ve always got people breathing down your neck, haven’t you? Where is the result for this? The consultant’s coming, and I need this out today.”*—HCPC Registered Academic AC1.

This comment demonstrated a perception of “them and us” between Biomedical Scientists and clinical staff, rather than demonstrating the importance of all individual roles within healthcare. It is interesting that the pressures felt by academic stakeholders were not reflected in the Biomedical Scientist group.

#### The Multi-Disciplinary Team (MDT)

Participants also expressed that Biomedical Scientists often felt underappreciated within the wider healthcare system, and that both patients and other clinical staff failed to understand the role.

*“I think because we’re hidden, like we’re in the labs and we don’t get any sort of patient face-to-face care or receive patients face-to-face, so I think a lot of people forget that we are there, and we do help in the decision making.”*—Biomedical Science BS5.

As part of this, participants also discussed the importance of team-working and recognised that healthcare professionals must work together to support patient care.

*“If this was a member of my family that was being tested, would I be happy just leaving it like ‘I’m doing my job OK’? If we all work together as a team then, like everyone that’s involved in the patient pathway, then it will, I think, achieve a better outcome for the patient.”*—Student S2.

The “behind the scenes” nature of the Biomedical Scientist role was also discussed by participants.

*“People don’t always appreciate that there’s a laboratory behind that and the extent of the work, and, even when it does come into the lab, it’s not always a case of just putting a sample on an analyser and pressing a button and 5 minutes later you get the results.”*—Biomedical Scientist BS4.

The hidden nature of the role was perceived to provide a challenge when determining whether other healthcare professionals and patients understood the complexities of the role. Within the wider MDT, the failure to understand the intricacies of the role can have implications for how the role is perceived externally and can result in frustrations due to not understanding why certain policies and procedures are in place.

### Round 2

Statements scored by the participants are presented as paired positive and negative statements to aid data interpretation (Tables 4–7), but statements were randomised when shared with the participants to prevent bias. Consensus level is presented according to participant group and overall. The consensus statements used in round 2 of the study are tabulated and colour-coded to aid interpretation. Red shading represents achievement of consensus disagreement, whilst green shading represents consensus agreement and grey shading represents a failure to achieve consensus. Any statements where participants failed to provide a response are also recorded. These omitted responses may result from a participant accidentally failing to provide an answer to the statement, or from a deliberate choice as the participant deemed they were unable to express an opinion on a particular topic.

**TABLE 4:** Consensus levels for each of the paired statements for putting the patient first across all participant groups. Fields highlighted in green demonstrate achievement of an agree consensus, fields highlighted in red demonstrate achievement of a disagree consensus and fields highlighted in grey demonstrate that the 70% consensus level was not achieved.

Positive statements	Students (*n* = 7)	Biomedical scientists (*n* = 4)	Academics (*n* = 5)	Overall (*n* = 16)	Negative statements	Students (*n* = 7)	Biomedical scientists (*n* = 4)	Academics (*n* = 5)	Overall (*n* = 16)
Putting the patient at the centre									
2. Biomedical Scientists work in the best interests of the patient at all times	6/7 (85.7%) Agree	4/4 (100%) Agree	4/5 (80%) Agree	14/16 (87.5%) Agree	11. The desire to work in the best interests of the patient at all times is not shared by all Biomedical Scientists	1/7 (14.2%) Agree	2/4 (50%) Agree	3/5 (60%) Agree	6/16 (37.5%) Agree
	1/7 (14.2%) Neutral	-	-	1/16 (6.25%) Neutral		3/7 (42.9%) Neutral	-	1/5 (20%) Neutral	4/16 (25%) Neutral
	-	-	1/5 (20%) Disagree	1/16 (6.25%) Disagree		3/7 (42.9%) Disagree	2/4 (50%) Disagree	1/5 (20%) Disagree	6/16 (37.5%) Disagree
25. Despite the lack of proximity to the patient, Biomedical Scientists put the patient at the centre of what they do	6/7 (85.7%) Agree	4/4 (100%) Agree	2/5 (40%) Agree	12/16 (75%) Agree	3. It is easier for staff providing direct patient care to prioritise patient outcomes due to their proximity to the patient	5/7 (71.4%) Agree	2/4 (50%) Agree	4/5 (80%) Agree	11/16 (68.75%) Agree
	-	-	1/5 (20%) Neutral	1/16 (6.25%) Neutral		1/7 (14.2%) Neutral	-	-	1/16 (6.25%) Neutral
	1/7 (14.2%) Disagree	-	2/5 (40%) Disagree	3/16 (18.75%) Disagree		1/7 (14.2%) Disagree	(2/4) 50% Disagree	1/5 (20%) Disagree	4/16 (25%) Disagree
35. A Biomedical Scientist should recognise the importance of each individual sample by considering the needs of the patient behind each sample	6/7 (85.7%) Agree	4/4 (100%) Agree	5/5 (100%) Agree	15/16 (93.75%) Agree	43. It’s possible for a Biomedical Scientist to do their job well without considering the needs of the patient	2/7 (28.6%) Agree	-	2/5 (40%) Agree	4/16 (25%) Agree
	-	-	-	-		2/7 (28.6%) Neutral	-	1/5 (20%) Neutral	3/16 (18.75%) Neutral
	1/7 (14.2%) Disagree	-	-	1/16 (6.25%) Disagree		3/7 (42.9%) Disagree	4/4 (100%) Disagree	2/5 (40%) Disagree	9/16 (56.25%) Disagree
42. By focusing upon the outcome of the individual patient, the Biomedical Scientist achieves greater job satisfaction	6/7 (85.7%) Agree	4/4 (100%) Agree	5/5 (100%) Agree	15/16 (93.75%) Agree	26. Job satisfaction is not determined by the perceived importance of patients within the Biomedical Scientist role	3/7 (42.9%) Agree	-	1/5 (20%) Agree	4/16 (25%) Agree
	1/7 (14.3%) Neutral	-	-	1/16 (6.25%) Neutral		2/7 (28.6%) Neutral	-	1/5 (20%) Neutral	3/16 (18.75%) Neutral
	-	-	-	-		2/7 (28.6%) Disagree	4/4 (100%) Disagree	3/5 (60%) Disagree	9/16 (56.25%) Disagree
36. Biomedical Scientists are able to draw upon their own personal experiences as a patient or carer which allows them to empathise with the patient	6/7 (85.7%) Agree	4/4 (100%) Agree	2/5 (40%) Agree	12/16 (75%) Agree	4. Biomedical Scientists do not require empathy for patients to do their job effectively	2/7 (28.6%) Agree	-	1/5 (20%) Agree	2/16 (12.5%) Agree
	1/7 (14.3%) Neutral	-	2/5 (40%) Neutral	3/16 (18.75%) Neutral		1/7 (14.3%) Neutral	-	-	2/16 (12.5%) Neutral
	-	-	1/5 (20%) Disagree	1/16 (6.25%) Disagree		4/7 (57.1%) Disagree	4/4 (100%) Disagree	4/5 (80%) Disagree	12/16 (75%) Disagree
Key	Consensus (>70%) reached agree or strongly agree				Consensus (>70%) reached Disagree or strongly disagree	Failed to reach consensus			

### Round Two Findings to Support the Presence of a Theory-Practice Gap

Of the 44 consensus statements that were presented to the stakeholders in the 2nd round, only 45.5% of statements achieved consensus. The consensus level was highest in the Biomedical Scientist group, where 72.7% of statements achieved the 70% consensus level. However, in the student group, only 54.5% of statements achieved the 70% consensus level. . In the academic group, consensus level was lowest with only 40.9% of statements reaching the 70% consensus level. This demonstrated a divergence of opinion across the stakeholders. However, it is important to note that both PSRB representatives were lost in round 2 of the study.

#### Putting the Patient First

There were several statements which demonstrated the emergence of a theory-practice gap in both the academic and student groups when responses were compared with the Biomedical Scientist group. Those statements which relate to putting the patient first are presented in Table 4. One important finding was that for statement 11, “The desire to work in the best interests of the patient at all times is not shared by all Biomedical Scientists” did not reach consensus in any of the stakeholder groups. However, the positive version of the statement that “Biomedical Scientists work in the best interests of the patient at all times” reached consensus overall and in all stakeholder groups. As outlined in the limitations section, the phrasing of these two statements may have impacted upon the consensus rate as the statements were not a perfect pair.

Most participants agreed with statement 3, “Despite a lack of proximity to the patient, Biomedical Scientists put the patient at the centre of what they do.” However, this statement failed to reach consensus amongst the academic stakeholders with two participants disagreeing with this statement. This suggests that some stakeholders felt that patients were not considered to be a central part of the Biomedical Scientist role. This was further supported by statement 43, “It is possible for a Biomedical Scientist to do their job well without considering the needs of the patient” which exhibited divergence of opinion. This demonstrates that some individuals felt that a Biomedical Scientist could do their role well without focusing upon the importance of the patient, which is a key finding.

Statement 36, “Biomedical Scientists are able to draw upon their own personal experiences as a patient or carer which allows them to empathise with the patient,” failed to achieve consensus in the academic group, and one participant disagreed with this statement. The alternative version of the statement, (number 4), “Biomedical Scientists do not require empathy for patients to do their job effectively” failed to reach consensus in the student group. This supports the presence of a theory-practice gap in both the student and academic groups.

#### Professional Registration and Accreditation

The theory-practice gap was further evidenced when discussing professional responsibilities and the importance of laboratory accreditation and professional registration for Biomedical Scientists. Table 5 presents the key statements that indicate a theory-practice gap in both the academic and student groups related to professional registration and accreditation.

**TABLE 5:** Consensus levels for each of the paired statements for professional registration and accreditation across all participant groups. Fields highlighted in green demonstrate achievement of an agree consensus, fields highlighted in red demonstrate achievement of a disagree consensus and fields highlighted in grey demonstrate that the 70% consensus level was not achieved.

Positive statements	Students (*n* = 7)	Biomedical scientists (*n* = 4)	Academics (*n* = 5)	Overall (*n* = 16)	Negative statements	Students (*n* = 7)	Biomedical scientists (*n* = 4)	Academics (*n* = 5)	Overall (*n* = 16)
Safety of the patient, subtheme c: minimising harm									
27. The Biomedical Scientist plays an essential role in patient safety	5/7 (71.4%) Agree	4/4 (100%) Agree	5/5 (100%) Agree	14/16 (87.5%) Agree	45. It is not within the Biomedical Scientist’s remit to question clinical decisions that put the patient at risk	2/7 (28.6%) Agree	**-**	**-**	2/16 (12.5%) Agree
	2/7 (28.6%) Neutral	-	-	2/16 (12.5%) Neutral		2/7 (28.6%) Neutral	-	-	2/16 (12.5%) Neutral
	-	-	-	-		3/7 (42.9%) Disagree	4/4 (100%) Disagree	5/5 (100%) Disagree	12/16 (75%) Disagree
13. Biomedical Scientists must question inappropriate clinical decisions	4/7 (57.1%) Agree	4/4 (100%) Agree	5/5 (100%) Agree	13/16 (81.25%) Agree	5. Patient safety is the responsibility of others in the clinical team and it is not appropriate for a Biomedical Scientist to question their decision making	1/7 (14.3%) Agree	-	-	1/16 (6.25%) Agree
	2/7 (28.6%) Neutral	-	-	2/16 (12.5%) Neutral		1/7 (14.3%) Neutral	-	-	1/16 (6.25%) Neutral
	1/7 (14.3%) Disagree	-	-	1/16 (6.25%) Disagree		5/7 (71.4%) Disagree	4/4 (100%) Disagree	5/5 (100%) Disagree	14/16 (87.5%) Disagree
28. Statutory HCPC registration for Biomedical Scientists provides confidence for patients and others in the clinical team about the quality of the Biomedical Scientist’s practice	5/7 (71.4%) Agree	4/4 (100%) Agree	4/5 (80%) Agree	13/16 (81.25%) Agree	14. Statutory registration for Biomedical Scientists has no meaning to patients and others in the clinical team as they lack an understanding of the role	5/7 (71.4%) Agree	2/4 (50%) Agree	3/5 (60%) Agree	10/16 (62.5%) Agree
	2/7 (28.6%) Neutral	-	-	2/16 (12.5%) Neutral		1/7 (14.3%) Neutral	-	1/5 (20%) Neutral	2/16 (12.5%) Neutral
	-	-	1/5 (20%) Disagree	1/16 (6.25%) Disagree		1/7 (14.3%) Disagree	2/4 (50%) Disagree	1/5 (20%) Disagree	4/16 (25%) Disagree
37. Statutory registration with the Health and Care Professions Council (HCPC) ensures that Biomedical Scientists feel empowered to make autonomous decisions	2/7 (28.6%) Agree	3/4 (75%) Agree	2/5 (40%) Agree	7/16 (43.75%) Agree	15. Biomedical Scientists are not required to make autonomous decisions	2/7 (28.6%) Agree	-	1/5 (20%) Agree	3/16 (18.75%) Agree
	5/7 (71.4%) Neutral	-	1/5 (20%) Neutral	6/16 (37.5%) Neutral		1/7 (14.3%) Neutral	-	1/5 (20%) Neutral	2/16 (12.5%) Neutral
	-	1/4 (25%) Disagree	2/5 (20%) Disagree	3/16 (18.75%) Disagree		4/7 (57.1%) Disagree	4/4 (100%) Disagree	3/5 (60%) Disagree	11/16 (68.75%) Disagree
Key	Consensus (>70%) reached agree or strongly agree				Consensus (>70%) reached Disagree or strongly disagree	Failed to reach consensus			

Statement 45, “it is not within the Biomedical Scientist’s remit to question clinical decisions that put the patient at risk,” failed to achieve consensus in the student group, with 2 participants agreeing with this statement. This was further demonstrated in the student group in response to statement 13, “Biomedical Scientists must question inappropriate clinical decisions.” which did not achieve consensus amongst the student stakeholders, with one student participant disagreeing with this statement. These responses demonstrate a poor understanding of the Biomedical Scientist role in ensuring patient safety and advocating for the patient in the student group.

Statement 37, “Statutory registration with the Health and Care Professions Council (HCPC) ensures that Biomedical Scientists feel empowered to make autonomous decisions,” achieved consensus agreement amongst the Biomedical Scientist stakeholders only. Interestingly, amongst the academic participants, 2 stakeholders disagreed with this statement. The negative form of this statement (number 15), “Biomedical Scientists are not required to make autonomous decisions” reached a disagree consensus in the Biomedical Scientist group only. In response to this statement, 2 students and 1 academic agreed with the statement. This further supports the presence of a theory-practice gap in both the academic and student groups.

#### Pressures of the Role

This theme was identified in the academic group only. Statement 17, “the pressures of the Biomedical Scientist role do not detract from the importance of the individual patient,” achieved consensus agreement in the student group only. One academic participant omitted their response to this statement. This is shown in Table 6. The opposing statement (number 29), “other pressures of the Biomedical Scientist role detract from the importance of the individual patient” did not reach consensus in any of the stakeholder groups but 60% of academic participants agreed with this statement.

**TABLE 6:** Consensus levels for each of the paired statements for pressures of the role across all participant groups. Fields highlighted in green demonstrate achievement of an agree consensus, fields highlighted in red demonstrate achievement of a disagree consensus and fields highlighted in grey demonstrate that the 70% consensus level was not achieved.

Positive statements	Students (*n* = 7)	Biomedical scientists (*n* = 4)	Academics (*n* = 5)	Overall (*n* = 16)	Negative statements	Students (*n* = 7)	Biomedical scientists (*n* = 4)	Academics (*n* = 5)	Overall (*n* = 16)
Pressures of the role									
17. The pressures of the Biomedical Scientist role do not detract from the importance of the individual patient	6/7 (85.7%) Agree	2/4 (50%) Agree	1/5 (20%) Agree	9/16 (56.25%) Agree	29. Other pressures of the Biomedical Scientist role detract from the importance of the individual patient	1/7 (14.3%) Agree	-	3/5 (60%) Agree	4/16 25% Agree
	1/7 (14.3%) Neutral	2/4 (50%) Neutral	1/5 (20%) Neutral	4/16 (25%) Neutral		3/7 (42.9%) Neutral	2/4 (50%) Neutral	1/5 (20%) Neutral	6/16 (37.5%) Neutral
	-	-	2/5 (40%) Disagree	2/16 (12.5%) Disagree		3/7 (42.9%) Disagree	2/4 (50%) Disagree	1/5 (20%) Disagree	6/16 (37.5%) Disagree
	-	-	1/5 (20%) Omitted	1/16 (6.25%) Omitted		-	-	-	-
Key	Consensus (>70%) reached agree or strongly agree				Consensus (>70%) reached disagree or strongly disagree	Failed to reach consensus			

#### The Multi-Disciplinary Team

There was further evidence of the theory-practice gap with regards to the Biomedical Scientist role within the MDT. Statement 25, “Despite the lack of patient contact, Biomedical Scientists can focus upon achieving outcomes for each individual patient,” failed to reach consensus amongst the academic stakeholders and one participant in this group disagreed with the statement. The responses associated with the MDT are shown in Table 7. Statement 20, “the nature of the Biomedical Scientist role makes it difficult for them to feel part of the healthcare team,” reached consensus agreement in the Biomedical Scientist group only. This suggests that a real-world understanding of the Biomedical Scientist role may be lacking in both the academic and student groups.

**TABLE 7:** Consensus levels for each of the paired statements for the MDT across all participant groups. Fields highlighted in green demonstrate achievement of an agree consensus, fields highlighted in red demonstrate achievement of a disagree consensus and fields highlighted in grey demonstrate that the 70% consensus level was not achieved.

Positive statements	Students (*n* = 7)	Biomedical scientists (*n* = 4)	Academics (*n* = 5)	Overall (*n* = 16)	Negative statements	Students (*n* = 7)	Biomedical scientists (*n* = 4)	Academics (*n* = 5)	Overall (*n* = 16)
The multi-disciplinary team									
25. Despite the lack of patient contact, Biomedical Scientists can focus upon achieving outcomes for each individual patient	7/7 (100%) Agree	4/4 (100%) Agree	3/5 (60%) Agree	14/16 (87.5%) Agree	38. Lack of patient contact makes it difficult for a Biomedical Scientist to recognise their role within an individual patient’s outcomes	2/7 (28.9%) Agree	2/4 (50%) Agree	3/5 (60%) Agree	7/16 (43.75%) Agree
	-	-	1/5 (20%) Neutral	1/16 (6.25) Neutral		2/7 (28.9%) Neutral	-	-	2/16 (12.5%) Neutral
	-	-	1/5 (20%) Disagree	1/16 (6.25%) Disagree		3/7 (42.9%) Disagree	2/4 (50%) Disagree	2/5 (40%) Disagree	7/16 (43.75%) Disagree
47. Biomedical Scientists are well integrated within the healthcare team, despite a lack of patient contact	1/7 (14.3%) Agree	1/4 (25%) Agree	-	2/16 (12.5%) Agree	20. The nature of the Biomedical Scientist role makes it difficult for them to feel part of the healthcare team	2/7 (28.6%) Agree	4/4 (100%) Agree	3/5 (60%) Agree	9/16 (56.25%) Agree
	2/7 (28.6%) Neutral	1/4 (25%) Neutral	1/5 (20%) Neutral	4/16 (25%) Neutral		2/7 (28.6%) Neutral	-	1/5 (20%) Neutral	3/16 (18.75%) Neutral
	4/7 (57.1%) Disagree	2/4 (50%) Disagree	4/5 (80%) Disagree	10/16 (62.5%) Disagree		2/7 (28.6%) Disagree	-	1/5 (20%) Disagree	3/16 (18.75%) Disagree
	-	-	-	-		1/7 (14.3%) Omitted	-	-	1/16 (6.25%) Omitted
21. The role of the Biomedical Scientist in healthcare is well understood by other healthcare professionals	1/7 (14.3%) Agree	1/4 (25%) Agree	2/5 (40%) Agree	4/16 (25%) Agree	31. The role of the Biomedical Scientist in healthcare is poorly understood by other healthcare professionals	7/7 (100%) Agree	3/4 (75%) Agree	3/5 (60%) Agree	13/16 (81.25%) Agree
	1/7 (14.3%) Neutral	1/4 (25%) Neutral	-	2/16 (12.5%) Neutral		-	1/4 (25%) Neutral	-	1/16 (6.25%) Neutral
	5/7 (71.4%) Disagree	2/4 (50%) Disagree	3/5 (60%) Disagree	10/16 (62.5%) Disagree		-	-	2/5 (40%) Disagree	2/16 (12.5%) Disagree
8. Biomedical Scientists are regarded as being an essential part of the multi-disciplinary team by the wider healthcare team	4/7 (57.1%) Agree	1/4 (25%) Agree	2/5 (40%) Agree	7/16 (43.75%) Agree	32. Biomedical Scientists are not considered part of the multi-disciplinary team by the wider healthcare team	3/7 (42.9%) Agree	2/4 (50%) Agree	3/5 (60%) Agree	8/16 (50%) Agree
	1/7 (14.3%) Neutral	1/4 (25%) Neutral	-	2/16 (12.5%) Neutral		3/7 (42.9%) Neutral	-	-	3/16 (18.75%) Neutral
	2/7 (28.6%) Disagree	2/4 (50%) Disagree	3/5 (60%) Disagree	7/16 (43.75%) Disagree		1/7 (14.3%) Disagree	2/4 (50%) Disagree	2/5 (40%) Disagree	5/16 (31.25%) Disagree
33. Biomedical Scientists must recognise when referral to a consultant or member of the medical team is required to ensure the best outcome for a patient	5/7 (71.4%) Agree	4/4 (100%) Agree	5/5 (100%) Agree	14/16 (87.5%) Agree	40. Biomedical Scientists are not responsible for referring a patient case for a consultant to review	1/7 (14.3%) Agree	-	1/5 (20%) Agree	2/16 (12.5%) Agree
	2/7 (28.9%) Neutral	-	-	2/16 (12.5%) Neutral		2/7 (28.6%) Neutral	-	1/5 (20%) Neutral	3/16 (18.75%) Neutral
	-	-	-	-		3/7 (42.9%) Disagree	4/4 (100%) Disagree	3/5 (60%) Disagree	10/16 (62.5%) Disagree
	-	-	-	-		1/7 (14.3%) Omitted	-	-	1/16 (6.25%) Omitted
34. The technical and analytical nature of the Biomedical Scientist role does not detract from the importance of the individual patient	4/7 (57.1%) Agree	4/4 (100%) Agree	4/5 (80%) Agree	12/16 (75%) Agree	10. The technical and analytical nature of the Biomedical Scientist role makes it difficult to recognise the importance of the individual patient	2/7 (28.9%) Agree	-	2/5 (40%) Agree	4/16 (25%) Agree
	2/7 (28.9%) Neutral	-	-	2/16 (12.5%) Neutral		1/7 (14.3%) Neutral	-	1/5 (20%) Neutral	2/16 (12.5%) Neutral
	1/7 (14.3%) Disagree	-	1/5 (20%) Disagree	2/16 (12.5%) Disagree		4/7 (57.1%) Disagree	4/4 (100%) Disagree	2/5 (40%) Disagree	10/16 (62.5%) Disagree
41. Softer skills such as patient empathy are important aspects of the training and education of Biomedical Scientists	5/7 (71.4%) Agree	4/4 (100%) Agree	3/5 (60%) Agree	12/16 (75%) Agree	24. Biomedical Scientists do not require soft skills such as empathy, as their priority should be analysing specimens in a timely fashion	-	-	-	-
	1/7 (14.3%) Neutral	-	1/5 (20%) Neutral	2/16 (12.5%) Neutral		1/7 (14.3%) Neutral	-	1/5 (20%) Neutral	2/16 (12.5%) Neutral
	1/7 (14.3%) Disagree	-	1/5 (20%) Disagree	2/16 (12.5%) Disagree		6/7 (85.7%) Disagree	4/4 (100%) Disagree	4/5 (80%) Disagree	14/16 (87.5%) Disagree
Key	Consensus (>70%) reached agree or strongly agree				Consensus (>70%) reached disagree or strongly disagree	Failed to reach consensus			

There was divergence of opinion for how well the Biomedical Scientist role is understood by other professionals within healthcare. This was demonstrated by statement 21, “the role of the Biomedical Scientist in healthcare is well understood by other healthcare professionals” and statement 31, “the role of the Biomedical Scientist in healthcare is poorly understood by other healthcare professionals.” Statement 21 reached a disagree consensus amongst the student participants only. Statement 31 failed to achieve consensus in the academic group. This demonstrates that there was a divergence of opinion amongst the stakeholders with regards to the Biomedical Scientist role in the MDT.

This theory-practice gap was further reflected in statement 34, “the technical and analytical nature of the Biomedical Scientist role does not detract from the importance of the individual patient” which did not achieve consensus in the student group, with one student disagreeing with the statement. The negative version of the statement (number 10), “the technical and analytical nature of the Biomedical Scientist role makes it difficult to recognise the importance of the individual patient,” achieved consensus disagreement in the Biomedical Scientist group only. Amongst the student and academic stakeholders, two participants agreed with the statement, which conflicted with the findings from the Biomedical Scientist group.

Statement 41, “softer skills such as patient empathy are important aspects of the training and education of Biomedical Scientists,” failed to reach consensus amongst the academic stakeholders, and one participant disagreed with this statement. However, this response may have been made in error because the negative statement (number 24), “Biomedical Scientists do not require soft skills such as empathy as their priority should be analysing samples in a timely fashion” achieved consensus disagreement within all groups.

### Summary

Throughout both rounds of this study, a divergence of opinion was evident amongst the stakeholders, with more statements reaching consensus amongst the Biomedical Scientist group than in the student and academic groups. For some statements, there were individuals in the academic and student groups which expressed conflicting opinions to those of the Biomedical Scientist group. There was also evidence in both the student and academic groups that the Biomedical Scientist role and what was expected of this professional group was unclear in some cases. This demonstrates the existence of a theory-practice gap in both the academic and student groups, which has not previously been described.

## Discussion

Round 1 and 2 of this Delphi study demonstrated several key findings which are relevant to practice and enabled the initial research aims to be addressed. How the research has addressed these aims will now be outlined.

1. Is there consensus amongst stakeholders upon how the role of the Biomedical Scientist influences patient outcomes?

Stakeholders considered how the role of the Biomedical Scientist contributed towards achievement of patient outcomes and how these were considered important within the role. It was evident that many of the statements regarding the importance of the Biomedical Scientist role in achievement of patient outcomes demonstrated consensus amongst the stakeholders. However, greater divergence of opinion existed for whether Biomedical Scientists always work in the best interests of the patient, which is contrary to both professional and statutory guidelines [5, 6, 10].

2. Do stakeholders have a common understanding of how Biomedical Scientists might demonstrate that they are working to achieve patient outcomes?

Stakeholders felt that a Biomedical Scientist who was focused upon achieving patient outcomes demonstrated this through the high standard of their work, a professional attitude and adherence to professional guidelines and the HCPC standards [5]. These regulatory standards and professional guidelines were considered essential to standardise care and prevent harm.

3. How do stakeholders consider the importance of achieving patient outcomes within the Biomedical Scientist role?

Stakeholders recognised that achieving patient outcomes was evidenced through processing of samples to achieve a diagnosis and to initiate treatment pathways, which then supports other healthcare professionals to enable them to carry out their role. The stakeholders demonstrated consensus of opinion regarding the importance of the Biomedical Scientist role for contributing to patient outcomes through input into the MDT and this was considered an important consideration for entering the profession.

4. How do stakeholders recognise a Biomedical Scientist who is working to support the achievement of patient outcomes?

Participants identified this as a Biomedical Scientist that processes specimens accurately, ensuring that results are reported in a timely manner and, if necessary, are communicated within a clinically appropriate timeframe. Through these actions, Biomedical Scientists were seen as supporting the actions of other healthcare professionals through their role within the MDT. It was also considered essential for Biomedical Scientists to recognise when clinical referrals are necessary.

### The Theory-Practice Gap in the Education of Biomedical Scientists

This study has identified a theory-practice gap related to the education of Biomedical Scientists. The concept of a theory-practice gap has been extensively described in nursing and results from the difficulties in application of theoretical ideas delivered in an academic setting and the challenges of applying these to real-world professional practice [41]. Within the field of nursing, this gap has been found to be most significant for newly qualified professionals. This is because of the significant physical separation between a student’s academic studies and clinical practice, which is challenging when trying to relate theory to practice [41–43]. This is of greater significance in Biomedical Science, where only a small cohort of the students on the course will complete a healthcare laboratory placement.

This theory-practice gap has been described in several healthcare professions, including paramedic science [44], medicine [45], pharmacy [46] and extensively in nursing [41, 43, 47, 48]. However, this study has demonstrated for the first time that this gap exists within the Biomedical Scientist workforce. Now that this gap has been identified, it is necessary to develop strategies to overcome this gap and changes to the curriculum are likely to be required as a result.

This theory-practice gap amongst the stakeholders was identified due to divergence of opinion in both the academic and student groups when responses were compared to the Biomedical Scientist group. In addition, it became clear that more statements reached consensus in the Biomedical Scientist group than in the student and academic groups, with consensus level being lowest in the academic group. This demonstrates a difference between perceived understanding of the role in the academic and student groups. In the student responses, there was evidence of a larger degree of divergence of opinion, along with an increased number of responses recorded as “neither agree nor disagree” in round 2. This suggests that some of the student participants did not feel able to comment upon some aspects of the Biomedical Scientist role.

Graduates of a Biomedical Science programme aspire to a diverse range of careers, including teaching, research or further education. As not all graduates aspire to work as Biomedical Scientists this provides a challenge when designing the curriculum. Although placements at the end of the 2nd year of their award are open to all students, inevitably only a small number of placements in NHS laboratories are available on an annual basis. This prevents students from being exposed to key skills and gaining an in-depth knowledge of the Biomedical Scientist role.

Whilst the presence of this theory-practice gap amongst the student participants was perhaps not surprising as many student participants had not been exposed to working within a clinical laboratory, there was also evidence of the presence of a theory-practice gap amongst the academic group. Divergence in the responses in the academic group when compared to the Biomedical Scientist group demonstrated a lack of understanding of key aspects of the role. To obtain HCPC approval for a degree programme, it is necessary for a HCPC-registered Biomedical Scientist to take overall responsibility for oversight of the award. This is outlined in HCPC Standards of Education and Training (SET) 3.3 as follows: “The education provider must ensure that the person holding overall professional responsibility for the programme is appropriately qualified and experienced and, unless other arrangements are appropriate, on the relevant part of the Register” [49].

Unlike in other programmes allied to healthcare, not all academic staff teaching on the BSc Biomedical Science programmes have professional experience of working in NHS laboratories and being on the necessary professional register is not a requirement of all staff. Academics who lack personal experiences as a Biomedical Scientist may indirectly influence student responses through selection of curriculum content and delivery of taught materials. Practitioner involvement in professionally approved and accredited courses is essential for providing students with a realistic understanding of the role through instilling the skills required for professional practice into their students [50].

Participants demonstrated a divergence of opinion for several key statements, including when discussing whether all Biomedical Scientists worked in the best interests of the patient at all times. However, it is important to note that the phrasing of this statement to include “all Biomedical Scientists” rather than “Biomedical Scientists in general” may have contributed to this failure to reach consensus. This divergence was further evidenced when discussing whether it is possible for a Biomedical Scientist to do their job well without considering the needs of the patient, which also failed to reach consensus in the student and academic groups. This fails to comply with professional guidelines [10] and HCPC standards [5, 6], which require Biomedical Scientists to focus upon the best interests of patients and service users within their role. This suggests that a real-world understanding of the Biomedical Scientist role may be lacking in both the academic and student groups.

In Round one, one PSRB representative remarked that the understanding of key concepts related to the significance of achieving patient outcomes for Biomedical Scientists was “taken for granted.” It is important that guidelines and regulatory standards provided for healthcare professionals are clear, explicit and cannot be misinterpreted to guarantee patient safety [51, 52]. This statement by that PSRB representative demonstrates the rationale for this study and suggests the need for development of professional guidelines and standards which outline the skills and qualities expected of students and registered Biomedical Scientists. Unfortunately, in Round two, neither PSRB representative responded to the questionnaire, which is a recognised limitation.

Further evidence of the theory-practice gap was reflected when asked to score agreement regarding whether Biomedical Scientists are not required to make autonomous decisions. This reached consensus disagreement in the Biomedical Scientist group only. The statement showed divergence of opinion overall and in the student and academic groups. In response to this statement, two students and one academic demonstrated agreement, despite the disagree consensus in the Biomedical Scientist group. This further supports the theory-practice gap in the academic and student groups and suggests a poor understanding of the role of the Biomedical Scientist amongst these groups.

When considering whether Biomedical Scientists can focus upon achieving patient outcomes despite a lack of patient contact, an agree consensus was reached overall as well as in the student and Biomedical Scientist groups. However, this statement did not achieve consensus in the academic group, where one participant disagreed with the statement. This further supports the theory-practice gap and also contravenes the professional and statutory guidelines appropriate to the profession [5, 6, 10].

### Pressures of the Role

Considering whether the pressures of the Biomedical Scientist role detract from the importance of the patient, there was failure to achieve consensus in all the stakeholder groups. Although this did not achieve consensus, 60% of academic participants agreed with this statement. In Round one, several quotes demonstrated that the academic participants perceived the Biomedical Scientist role to be challenging with a “them and us” perception when discussing clinical staff. In the academic group, both workload pressures and time pressures were perceived to reduce the significance of achieving patient outcomes. In nursing, workload pressures are also recognised as detracting from high quality care and are known to impact patient safety [53, 54]. Amongst the academic stakeholders, the recognition of the pressures of the Biomedical Scientist role may have resulted in the decision to enter academia with a view to improving work-life balance. This has been recognised as motivation for nursing staff who pursue academic careers [55] but has not been described in relation to the Biomedical Scientist role.

### Social Desirability Bias

In Round two, there was an emerging pattern that positive versions of the statements achieved consensus more readily than negative versions. The data demonstrates that 13/22 (59.1%) of positive statements reached consensus whilst only 7/22 (31.8%) of negative statements reached consensus. This is a known limitation of researching using questionnaires as participants can agree with a statement whether it expresses their true opinion or not [33]. Participants are also at risk of social desirability bias, whereby they select responses that they believe are socially acceptable, even if this doesn’t represent their true opinion to prevent perceived judgement from the researcher [56, 57]. The use of paired attitude statements was designed to minimise the risk of social desirability bias by providing verification of the participant responses.

### Limitations of the Study

Whilst the study successfully addressed the research aims and questions, there are several limitations which will now be discussed. Recruitment of study participants yielded low participant numbers, particularly in the Biomedical Scientist group. Although Delphi studies do not have a specified minimum number of participants, it is generally accepted that at least 10–15 participants should be included in the panel [25, 39]. Unfortunately, due to low numbers of participants, it is necessary to recognise the limitations of drawing conclusions with such a small sample size [58]. This may have resulted from the challenges of conducting the research during a pandemic when laboratories were subject to staff absences and increased workloads [59]. Upon reflection, it would have been advantageous to seek an amendment from the ethics committees to approach several NHS Trusts for participants and to consider an additional stage of recruitment.

When analysing the Round 2 data, it was clear that for some of the attitude statements, the positive and negative versions were not perfect mirror images of each other. As a result, this may have caused some of the inconsistencies seen within the stakeholder responses. The fact that more positive statements achieved consensus than negative statements may have resulted from this ambiguity with the statement wording. This may have also been a factor in the satisficing observed in round 2 [33].

It was considered important to recruit PSRB individuals to participate in the study. Representatives of three PSRB organisations received an invitation to participate, but only a single organisation responded favourably. It was unfortunate that both PSRB representatives from the 1st Round were lost in the 2nd Round. It is necessary to include PSRB involvement in courses associated with specific professions to ensure that course content is appropriate and academic staff have the required knowledge to facilitate achievement of learning outcomes [60]. Recruitment of PSRB representatives to the study was essential to influence professional practice. As a result, the loss of both PSRB representatives in the Round 2 was an unfortunate limitation of the study.

Although the study explored the perceptions of several different stakeholders of the Biomedical Scientist role, it would have been beneficial to include patients and carers as a final stakeholder group. Unfortunately, time pressures prevented this key group from inclusion, and this is something that should be addressed in future research. A further limitation of the study was that the attitude statements were piloted with a group of 15 academic staff. In hindsight, this piloting exercise should have been carried out with a variety of stakeholder groups. As Delphi methodology is associated with a degree of attrition [25, 35], it was considered necessary to distribute the 2nd round questionnaire without undue delay. This meant that there was insufficient time to pilot the questionnaire with a range of stakeholders.

Stakeholders in the academic and student groups were recruited from a single academic organisation and Biomedical Scientist participants were recruited from a single NHS Trust. This represents a narrow scope for the study. Furthermore, the online survey which was distributed to the participants in the 2nd Round could have been shared with a wider audience of key stakeholders. This will be carried out as a 3rd Round of the Delphi study in the future to determine whether the findings of the study are more widely applicable. It would be worthwhile verifying the findings of this study across a range of NHS Trusts and a range of academic institutions by completing a further round of data collection to verify the understanding of a larger audience.

### Recommendations From the Study

• Changes to the professional guidelines and regulatory standards are required to include the addition of important concepts identified in the study. This includes the patient-focused elements of the Biomedical Scientist role, as well as empathy, the requirement to work in the best interests of the patient and how these requirements can be evidenced. The use of the term “service user” in the HCPC standards of proficiency [5, 6] may be unclear for students. Consequently, the term “service user” should be clearly defined within the context of patient care and the importance of putting the patient first.
• Course content for the BSc Biomedical Science programme should also discuss the role of the Biomedical Scientist in healthcare to adequately prepare students for practice and should be incorporated within a professional practice module for those students pursuing a career in a clinical diagnostic laboratory.
• The role of the Biomedical Scientist within the MDT was poorly understood, and participants perceived that the role was undervalued within healthcare. To gain recognition for the role within the MDT, it is necessary to promote and publicise the role externally and for students on Biomedical Science awards. This content should be delivered through collaboration involving both higher education and healthcare organisations.
• Biomedical Scientists need to be more integrated into the wider healthcare system to increase awareness of their professional knowledge and skills. To develop understanding of the Biomedical Scientist role as part of the MDT, MDT meetings should be attended by experienced Biomedical Scientists to gain external recognition of the complexities of the role. Although clinical staff attending MDT meetings can focus upon clinical aspects of a complex case, Biomedical Scientists can advise upon technical matters such as whether an existing sample is available for further testing or how long this would take. This opportunity will provide greater opportunity for Biomedical Scientists to focus upon patient outcomes within their role.
• Now that a theory-practice gap in the education of Biomedical Scientists has been identified, it is necessary to develop strategies to reduce the gap and these must be evaluated to ensure that graduates are better prepared for their role.
• In recognition of the wide range of potential careers of Biomedical Science graduates, it would be beneficial to distinguish between those students aspiring to a career in a diagnostic laboratory and those with other career aspirations. This would allow the cohort who wish to pursue careers in a diagnostic laboratory to receive more tailored and relevant degree content. However, this would be challenging to deliver as students are not always clear on their career aspirations early on in their degree.
• Academics delivering the BSc Biomedical Science programme without experience as Biomedical Scientists should be given the opportunity to explore this “real-world” setting to develop a better understanding of the intricacies of the Biomedical Scientist role. The patient-focused aspects of the course should be delivered by experienced practitioner lecturers who possess professional experience in this area. The content of the degree programme should also include an overview of the role of the Biomedical Scientist within the MDT. Students on Biomedical Science programmes should be better integrated with other students on professional health related courses to foster this understanding of the post-graduate role.

## Summary Table

### What Is Known About This Subject?

• Anecdotal evidence exists of the importance of patient outcomes for Biomedical Scientists, but this has not been documented.
• The role pathology services play within patient care pathways is well recognised.
• Students on BSc Biomedical Science programmes do not always have the opportunity to complete an integrated placement.

### What This Work Adds

• Provides evidence of a theory-practice gap relating to multi-disciplinary patient care and the role of Biomedical Scientists.
• Following identification of a gap, strategies for education and training are required to address this.
• The Biomedical Scientist role within the MDT is undervalued and poorly understood outside of the laboratory.

### Concluding Statement

This work represents an advance in biomedical science because the study has identified a theory-practice gap for the first time within Biomedical Scientist education.

## Data Availability

The original contributions presented in the study are included in the article/Supplementary Material, further inquiries can be directed to the corresponding author.
