# Supplementary material for: Identification of a Theory-Practice Gap in the Education of Biomedical Scientists
Source: Br J Biomed Sci. 2024 Jun 12;81:12629. doi: 10.3389/bjbs.2024.12629 (PMC11200117; doi:10.3389/bjbs.2024.12629)

JISC Online Surveys Questionnaire used in the 2^nd^ round of the study.

Page 1: Welcome


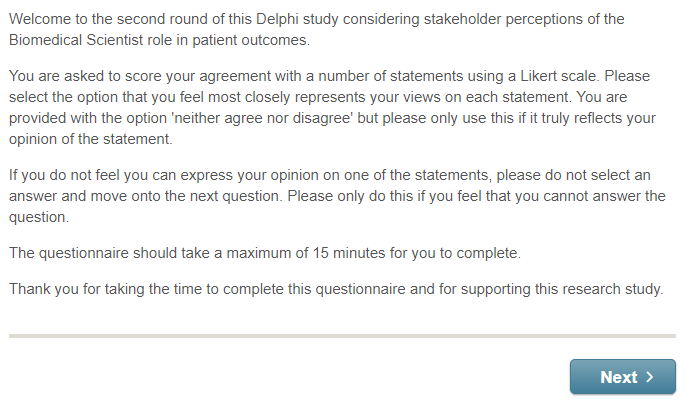


**Page 2: Participant group**


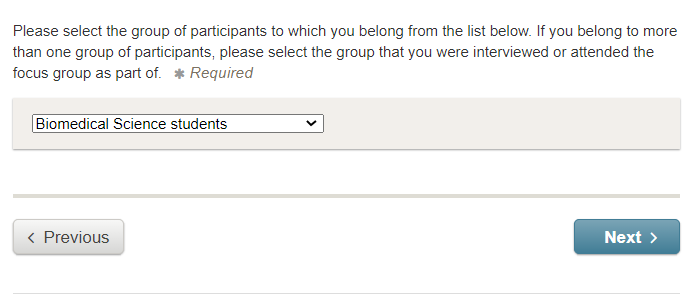


**Page 3: Questions 1-10**


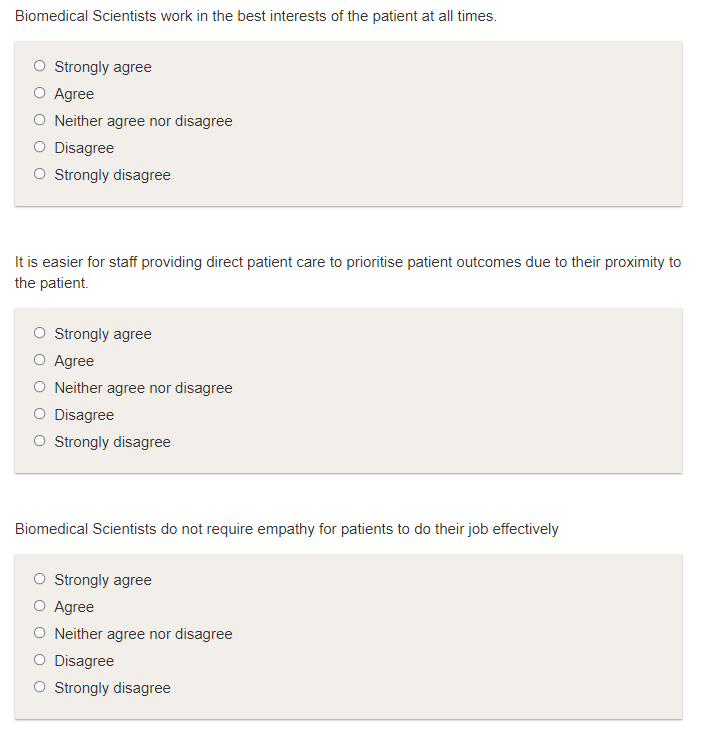


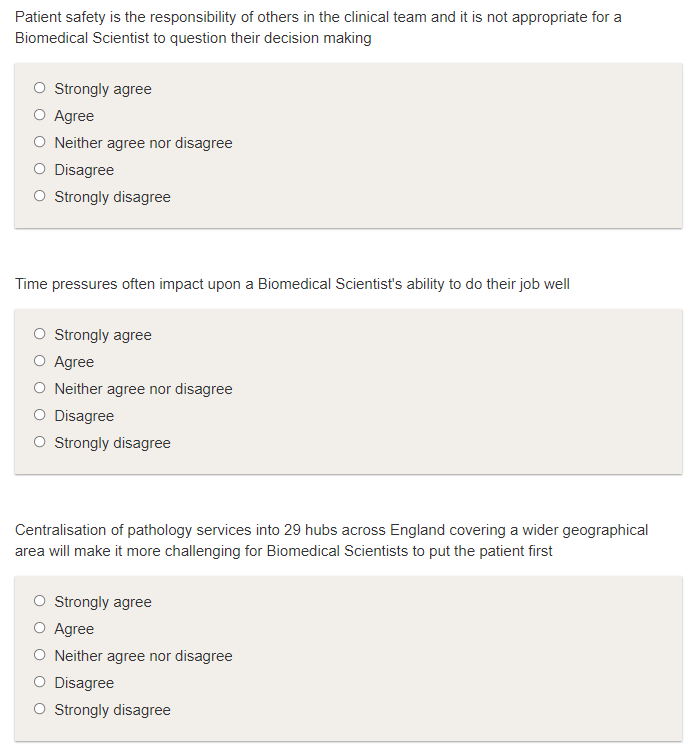


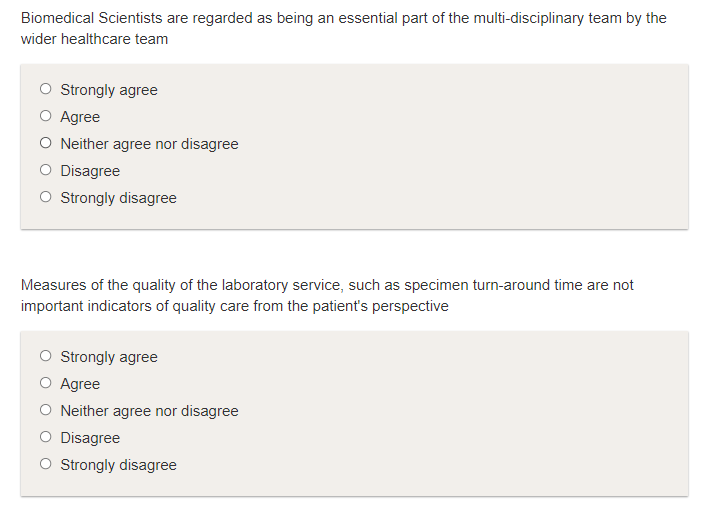


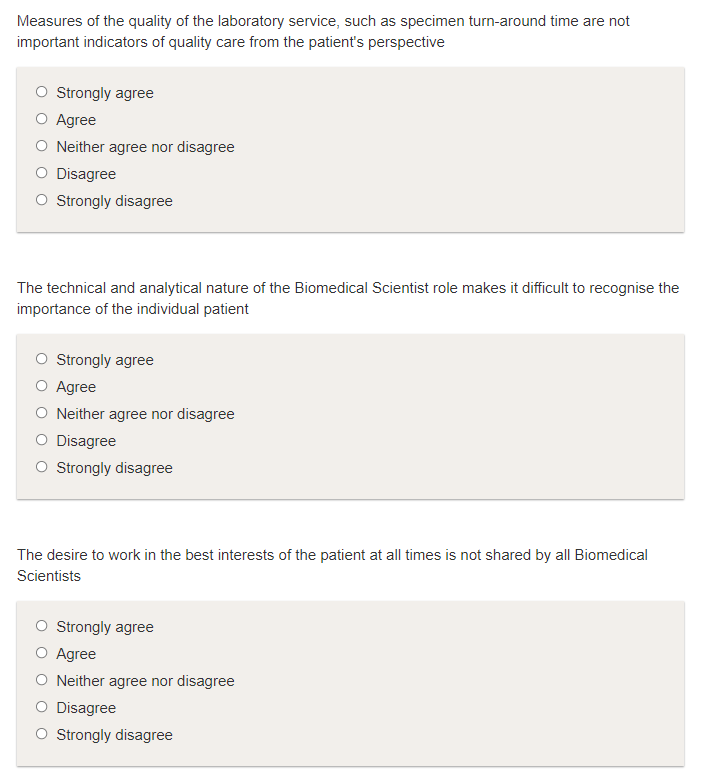


**Page 4: Questions 11-20**


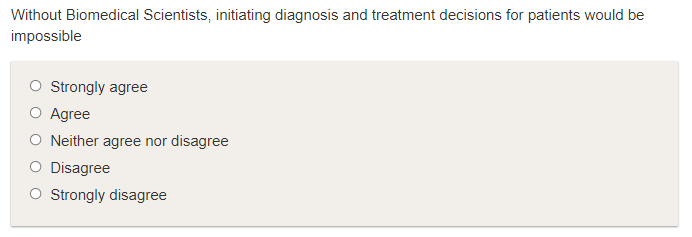


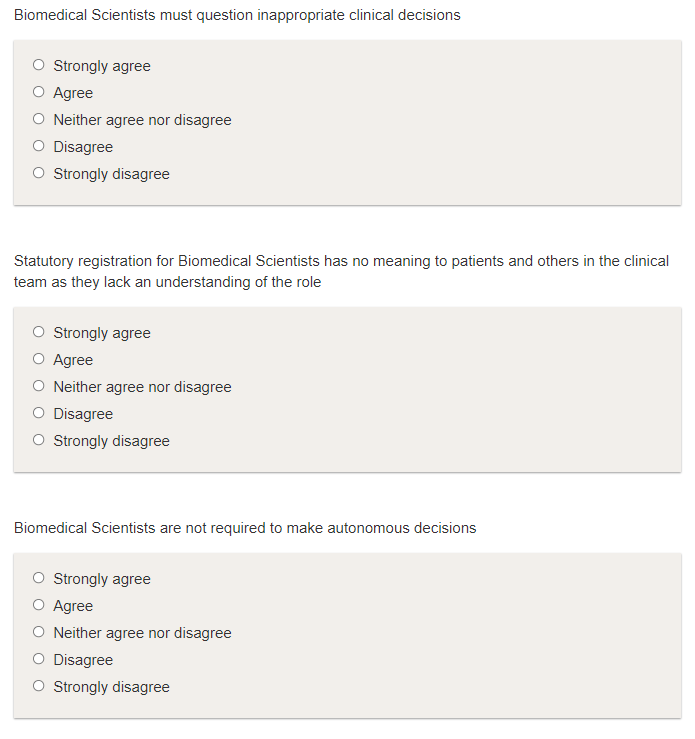


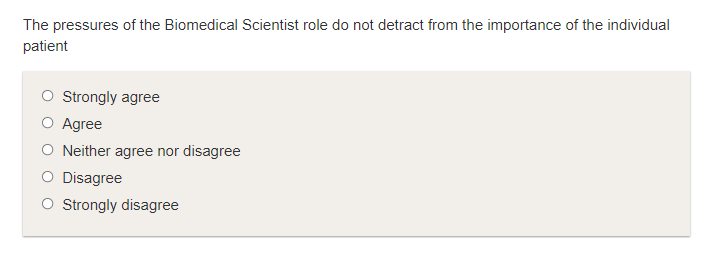


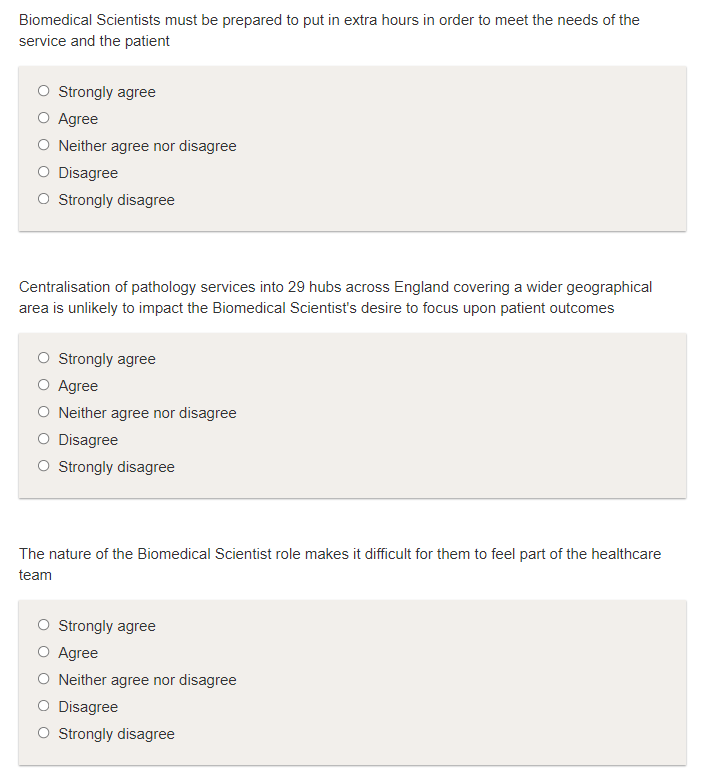


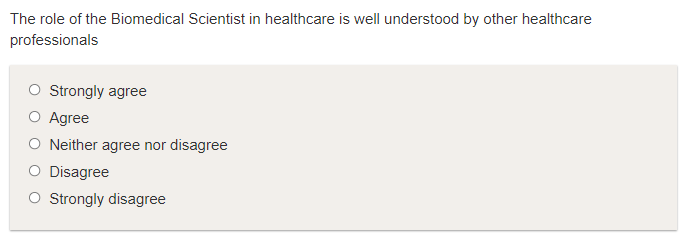


**Page 5: Questions 21-30**


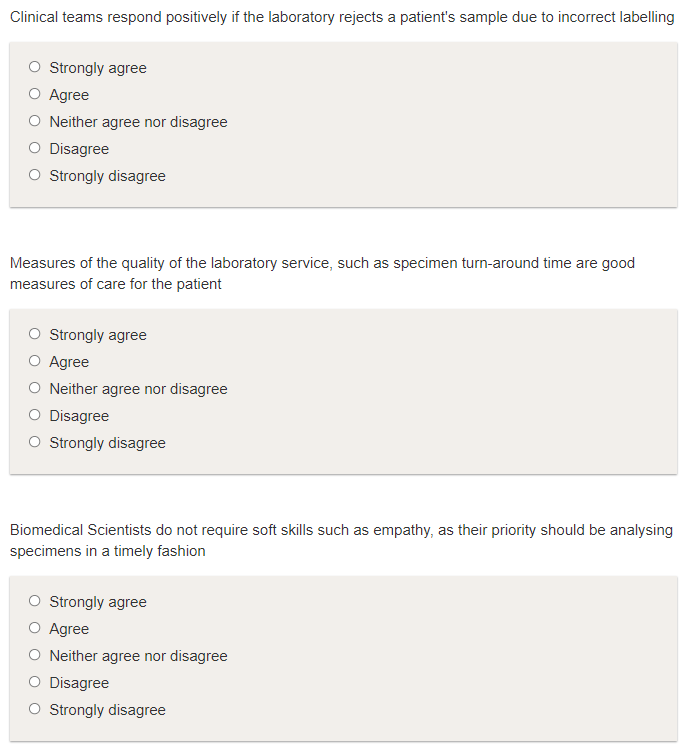


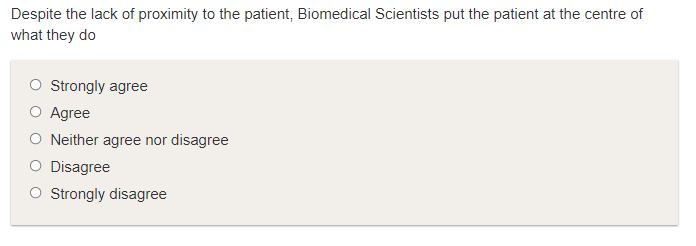


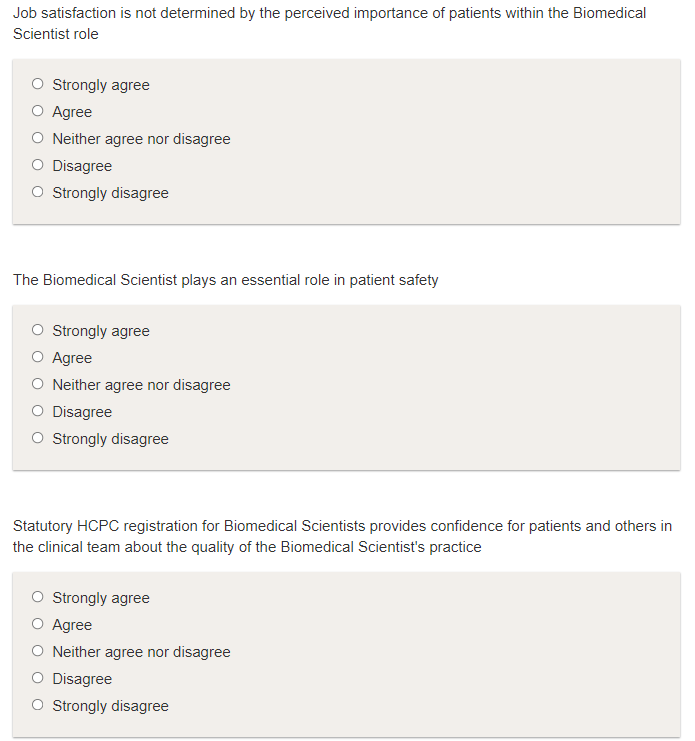


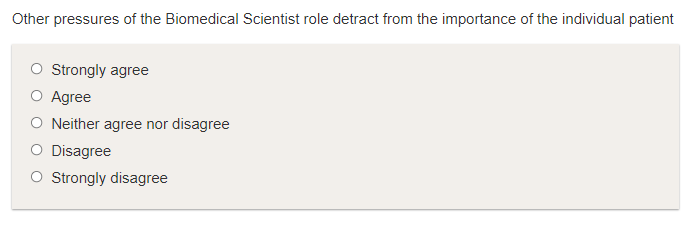


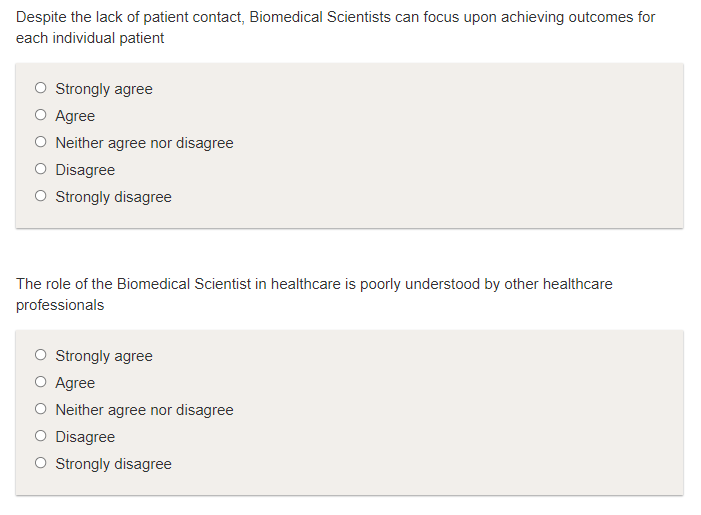


**Page 6: Questions 31-40**


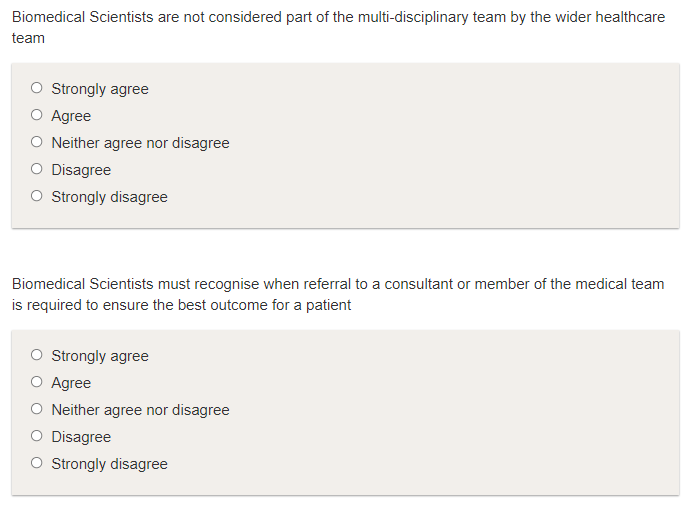


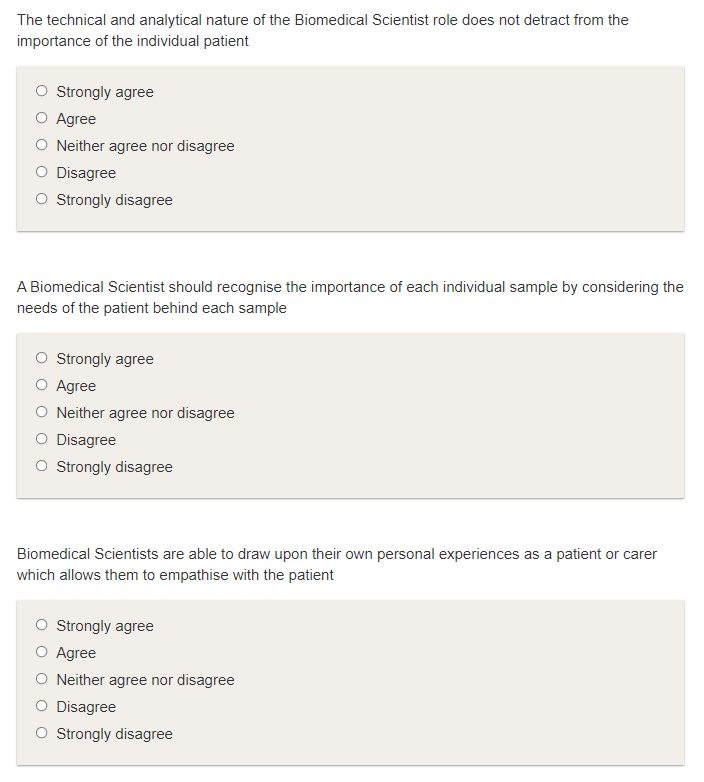


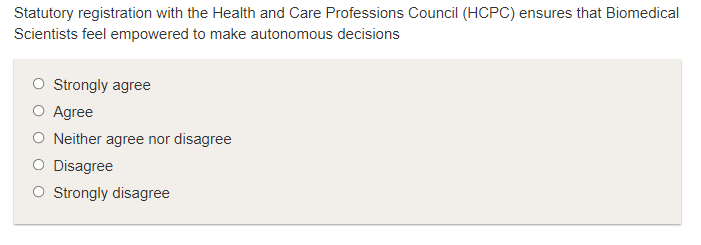


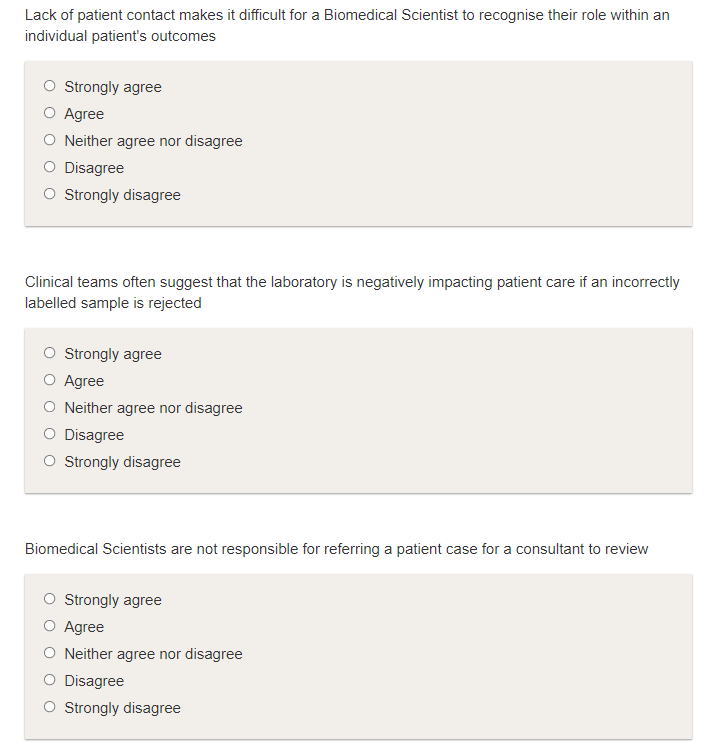


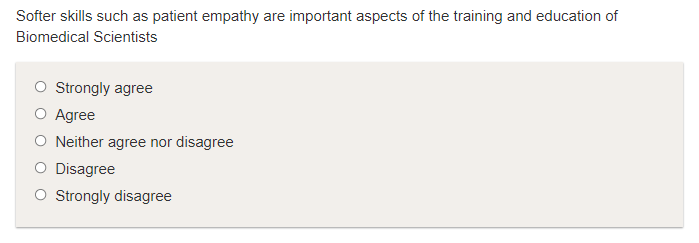


**Page 7: Questions 41-47**


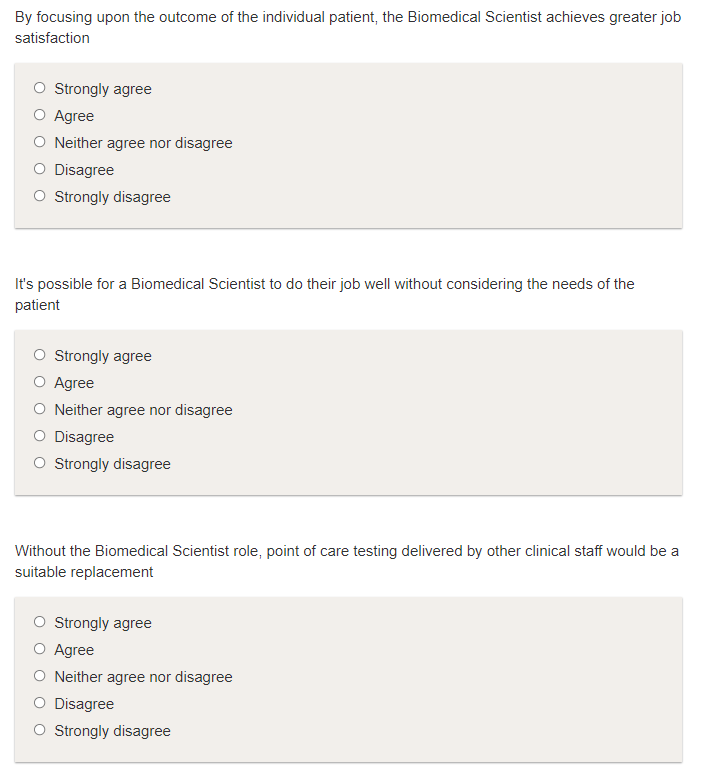


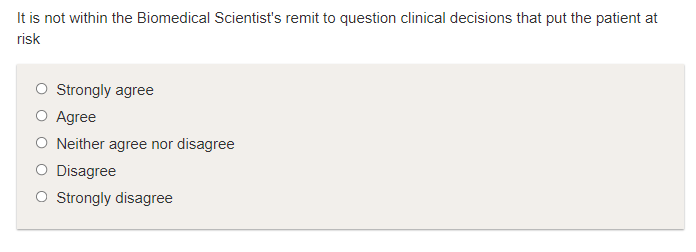


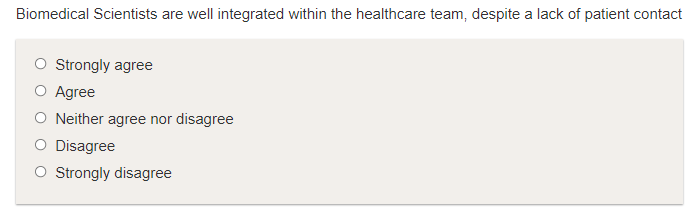


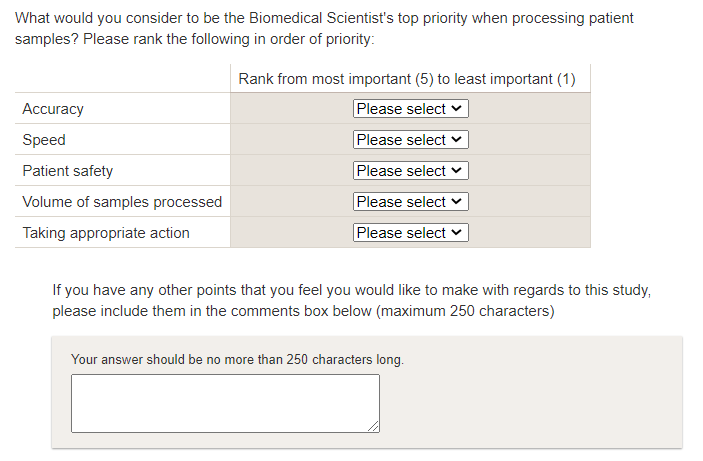

Supplement: Supplementary file 1 [file DataSheet2.docx]
