# Supplementary material for: Identification of a Theory-Practice Gap in the Education of Biomedical Scientists
Source: Br J Biomed Sci. 2024 Jun 12;81:12629. doi: 10.3389/bjbs.2024.12629 (PMC11200117; doi:10.3389/bjbs.2024.12629)
Supplement: Supplementary file 2 [file DataSheet1.docx]

**Presentation of the clinical vignette and any discussions that arise following the vignette.**

**Vignette:**

A 65-year-old woman presents to her GP complaining she feels tired all of the time and has no energy. She has noticed some bruising and bleeding when she brushes her teeth. The GP is concerned so requests urgent blood tests and sends an urgent biochemical profile, full blood count and coagulation screen to the local hospital. The results of the patient’s full blood count reveal a very low haemoglobin, high white blood cells with immature forms known as blasts, and dramatically low platelets which is the likely cause of her bruising and bleeding.

What do you perceive to be the Biomedical Scientist’s role in contributing to this patient’s outcomes? What would a good outcome be for this patient?

What role does the Biomedical Scientist play within a multi-disciplinary team in terms of achieving outcomes for this patient?

1. Participants will be asked what they feel the Biomedical Scientist’s role is within the vignette.
2. What does putting the patient at the centre of the Biomedical Scientist role mean to you? [Could you provide examples from your own practice of how you have done this?]
3. What do you think is the Biomedical Scientist’s role is achieving patient outcomes?
4. What do you understand by a Biomedical Scientist working to achieve patient outcomes?
5. The IBMS ‘Good Professional Practice in Biomedical Science’ (2019) states:

“Biomedical science professionals must exercise their professional duty of care and work within the legal, ethical and regulatory frameworks that govern and affect practice.”

What is the importance of these professional requirements and how can Biomedical Scientists achieve them?

1. What is the potential impact upon patient care if Biomedical Scientists are not focused upon patient outcomes?
2. How would you identify a Biomedical Scientist who is or isn’t focused upon achieving patient outcomes?
